# Motion Primitive Approach to Spacecraft Trajectory Design in a Multi-body System

**DOI:** 10.1007/s40295-023-00395-7

**Published:** 2023-09-11

**Authors:** Thomas R. Smith, Natasha Bosanac

**Affiliations:** Colorado Center for Astrodynamics Research, Smead Department of Aerospace Engineering Sciences, University of Colorado Boulder, 3775 Discovery Dr., Boulder, CO 80303 USA

**Keywords:** Cislunar space, Motion primitives, Initial guess construction, Rapid trajectory design, Multi-body gravitational systems

## Abstract

The increasing number and variety of spacecraft that are expected to operate within cislunar space and other multi-body gravitational environments throughout the solar system necessitates the continued development of strategies for rapid trajectory design and design space exploration. In the field of robotics, similar needs have been addressed using motion primitives that capture the fundamental building blocks of motion and are used to rapidly construct complex paths. Inspired by this concept, this paper leverages motion primitives to construct a framework for rapid and informed spacecraft trajectory design in a multi-body gravitational system. First, motion primitives of fundamental solutions, e.g., selected periodic orbits and their stable and unstable manifolds, are generated via clustering to form a discrete summary of segments of the phase space. Graphs of motion primitives are then constructed and searched to produce primitive sequences that form candidate initial guesses for transfers of distinct geometries. Continuous transfers are computed from each initial guess using multi-objective constrained optimization and collocation. This approach is demonstrated by constructing an array of geometrically distinct transfers between libration point orbits in the Earth-Moon circular restricted three-body problem with impulsive maneuvers.

## Introduction

A challenging aspect of spacecraft trajectory design in cislunar space and other multi-body systems is developing a systematic, rapid, and robust process for initial guess construction. The difficulty of constructing an initial guess depends on the complexity of the design space and the quality of an initial guess impacts the ability to recover a feasible solution. Even in a low-fidelity approximation of a multi-body gravitational environment, such as the circular restricted three-body problem (CR3BP), the solution space is complex and analytical solutions do not exist [33]. Consequently, initial guess construction may become a challenging and potentially time-consuming task for the trajectory designer, particularly when there are significant constraints derived from mission requirements or hardware parameters.

One existing approach to initial guess construction in multi-body systems leverages Poincaré mapping. First, fundamental solutions from low-fidelity models such as the CR3BP are computed. These fundamental solutions and/or a wider variety of trajectories are often manually examined using Poincaré maps that display their intersections with a hyperplane [13, 21]. Individual arcs are then manually selected, along with maneuver placement schemes, to form a discontinuous initial guess. A corrections and/or optimization scheme is then used to recover a continuous trajectory in the CR3BP or a higher fidelity model, subject to relevant constraints, parameters, and objectives. In this existing approach, two significant challenges emerge, particularly when designing maneuver-enabled, spatial trajectories: (1) visualizing and analyzing higher-dimensional datasets to manually select suitable arcs that form an initial guess for a nearby trajectory, and (2) manually constructing multiple geometrically distinct initial guesses to explore the solution space. Motivated by these challenges, Smith and Bosanac have recently introduced a motion primitive approach to spacecraft trajectory design in multi-body systems [30–32].

In the field of robotics, motion primitives have been used to construct complex paths. Wolek and Woolsey described a motion primitive as a “feasible trajectory that is used as a fundamental building block to construct more complex paths” [41]. This concept is often used in robotics to reduce the complexity of motion planning [22, 41]. As an example, Frazzoli et al. formed a finite library of trim and maneuver primitives for an aerial vehicle in a time invariant dynamical system [9]. In their work, a motion plan is defined as a sequence of concatenated motion primitives where a finite-state machine, denoted as a maneuver automaton, is represented as a graph and governs how primitives can be assembled into a sequence. Similarly, Grymin et al. reframed the motion planning problem as a graph search problem, a common technique in robotics and motion planning, by constructing a graph of reachable states in an environment connected by primitives from a precomputed library [12].

Inspired by their application to robotics, Smith and Bosanac have applied the concept of motion primitives to spacecraft trajectory design in a multi-body system. Specifically, we have formulated a consensus clustering procedure to numerically construct sets of motion primitives that summarize periodic orbit families and arcs along hyperbolic invariant manifolds based on geometry, stability, and energy in the Earth-Moon CR3BP [32]. We have then manually constructed sequences of motion primitives to produce coarse, primitive-based initial guesses that successfully enable the recovery of nearby natural and maneuver-enabled transfers between libration point orbits in the Earth-Moon CR3BP [30]. This paper builds upon our previous work by using graph theory to guide the primitive-based initial guess construction process for spacecraft trajectory design in the Earth-Moon CR3BP with impulsive maneuvers.

The utility of graph-based searches in initial guess construction within astrodynamics has been demonstrated by a variety of researchers. Tsirogiannis explored a graph-based methodology for designing impulsive transfers between periodic orbits in the CR3BP using Dijkstra’s algorithm [36]. Trumbauer and Villac developed an automated heuristic search-based framework for redesigning trajectories onboard a spacecraft in the CR3BP using precomputed dynamical structures, periapsis Poincaré maps, and the A* search algorithm [35]. Das-Stuart et al. constructed initial guesses for trajectories in the low-thrust enabled CR3BP using known dynamical structures, reinforcement learning, and Dijkstra’s algorithm [5]. Furthermore, Parrish leveraged a graph-based approach for computing optimal continuous-thrust trajectories in the two-body problem using the A* search algorithm [26]. More recently, Bruchko and Bosanac used probabilistic roadmap generation and Dijkstra’s algorithm to generate transfers between Lyapunov orbits in the CR3BP [3]. Although these contributions use distinct approaches for discretizing the solution space, they demonstrate the value of reframing the trajectory design problem as a discrete graph search problem.

This paper presents a motion primitive framework for rapid and informed trajectory design in the Earth-Moon CR3BP with impulsive maneuvers. First, sets of motion primitives are constructed to summarize the fundamental geometry, stability, and/or energy characteristics of members of individual periodic orbit families and arcs along their hyperbolic invariant manifolds in the CR3BP [32]. These sets, along with a small number of representative trajectories with similar characteristics, form a motion primitive library. A graph is then used to reflect the potential connectivity of these motion primitives in the library: primitives form the nodes of the graph with edges connecting them to their *k*-nearest neighbors and weighted to reflect their potential to produce a nearby continuous trajectory. This graph is constructed in two steps: (1) subgraphs are constructed to reflect the potential connectivity of motion primitives that summarize each individual orbit family or hyperbolic invariant manifold, and (2) these subgraphs are then connected using a modular high-level itinerary graph that reflects complete or partial information about the itinerary of the desired trajectory. The resulting motion primitive graph is then searched to produce distinct sequences of motion primitives that form initial guesses for transfers with distinct geometries. Continuous transfers with impulsive maneuvers are recovered from each initial guess using constrained, local optimization and collocation [30, 31]. This entire process is demonstrated by computing transfers of various geometries between Lyapunov and halo orbits near $L_1$ and $L_2$ in the Earth-Moon CR3BP. The result is a demonstration of an initial guess construction framework that uses motion primitives to generate candidate initial guesses for transfers of distinct geometries in cislunar space.

## Background

### Dynamical Model

The CR3BP is used to model the motion of a spacecraft of assumed negligible mass due to the point mass gravitational influences of the Earth and the Moon, with masses $M_1$ and $M_2$, respectively, and traveling on circular orbits about their barycenter [33]. A rotating reference frame is defined using an origin at the barycenter of the two primary bodies and axes $\{\varvec{{\hat{x}}}, \varvec{{\hat{y}}}, \varvec{{\hat{z}}}\}$: $\varvec{{\hat{x}}}$ is directed from the Earth to the Moon, $\varvec{{\hat{z}}}$ is aligned with the orbital angular momentum vector of the primary system, and $\varvec{{\hat{y}}}$ completes the right-handed triad [33]. In addition, quantities are often nondimensionalized using characteristic parameters for length ($l^*$), mass ($m^*$), and time ($t^*$): $l^*$ equals the assumed constant distance between the Earth and Moon, $m^*$ equals the total mass of the system, and $t^*$ produces a nondimensional period of the primary system equal to $2\pi$ [21, 33]. In the rotating frame, the nondimensional state of the spacecraft is then defined as $\varvec{x} = [x, y, z, {\dot{x}}, {\dot{y}}, {\dot{z}}]^{\text {T}}$ and the resulting equations of motion are written as

1 $$\begin{aligned} \ddot{x} = 2\dot{y} + \frac{\partial U^*}{\partial x} \hspace{15pt} \ddot{y} = -2\dot{x} + \frac{\partial U^*}{\partial y} \hspace{15pt} \ddot{z} = \frac{\partial U^*}{\partial z} \end{aligned}$$

where $U^* = 0.5(x^2 + y^2) + (1 - \mu )/r_1 + \mu /r_2$, $\mu = M_2/(M_1 + M_2)$, $r_1 = \sqrt{(x+\mu )^2 + y^2 + z^2}$, and $r_2 = \sqrt{(x-1+\mu )^2 + y^2 + z^2}$. In the Earth-Moon system, the mass ratio is $\mu \approx 0.01215$. Finally, this autonomous dynamical system admits an integral of motion, the Jacobi constant, equal to

2 $$\begin{aligned} C_J = 2U^* - \dot{x}^2 - \dot{y}^2 - \dot{z}^2 \end{aligned}$$

This quantity supplies insight into allowable regions of motion as well as heuristics for maneuver and trajectory design [21, 33].

### Computing Fundamental Solutions

Fundamental solutions in the CR3BP often support the construction of initial guesses for trajectories in a multi-body system. For instance, periodic and quasi-periodic orbits support identifying candidates for mission and staging orbits whereas their hyperbolic invariant manifolds may approximate natural transport mechanisms throughout the system [10, 17, 21]. Thus, this proof of concept for a primitive-based trajectory design framework focuses on using motion primitives that summarize selected families of periodic orbits and arcs along their stable and unstable manifolds. This subsection summarizes the methods used to compute these fundamental solutions in the CR3BP.

#### Periodic Orbits

In the CR3BP, a periodic orbit is a trajectory that repeats in the rotating frame and exists in a continuous family [21, 33]. In this paper, Lyapunov and halo orbits are computed numerically using a free variable and constraint vector formulation of multiple shooting [2, 21]. For Lyapunov orbits, an initial guess is generated using a stability analysis of a nearby equilibrium point; a bifurcation analysis of each family of Lyapunov orbits is used to generate an initial guess for a halo orbit [21, 33]. The initial guess is first discretized into several arcs with equal integration times. The states at the beginning of each arc, along with the common integration time, are assembled to form the free variable vector. Next, a constraint vector is defined to enforce state continuity between each arc as well as periodicity. The free variable vector is iteratively updated from an initial guess using Newton’s method and analytical derivatives of the constraints with respect to the free variables until the magnitude of the constraint vector is below $10^{-12}$; the result is a numerical approximation of a periodic orbit in the CR3BP [2, 21]. Pseudo-arclength continuation is then used to compute additional periodic orbits along each family [2, 19].

The stability of a periodic orbit supplies insight into the behavior of the nearby flow. Orbital stability is typically assessed using the eigenvalues of the monodromy matrix of a state along a periodic orbit [21]. The characteristics of two nontrivial and reciprocal eigenvalue pairs indicate the types of nearby motions: bounded trajectories exist near a periodic orbit with at least one pair of eigenvalues that lie on the unit circle in the complex plane whereas real eigenvalues indicate the existence of stable or unstable invariant manifolds. Each periodic orbit may be described by two stability indices $s_i$ for $i = [1,2]$, defined as the sum of the eigenvalues in each nontrivial, reciprocal pair [16].

#### Hyperbolic Invariant Manifolds

A trajectory along a stable manifold asymptotically approaches a periodic orbit in forward time whereas a trajectory along an unstable manifold asymptotically approaches the orbit in backward time [21]. In the absence of generalized analytical descriptions, an approximation of a stable or unstable half-manifold is typically computed numerically. First, an unstable periodic orbit is discretized into a set of states. At each state, $\varvec{x}_{{{\textbf {PO}}}}$, a perturbation of magnitude *d* is applied in the direction of a stable (or unstable) eigenvector, $\varvec{v}_{{\textbf {s}}/{\textbf {u}}}$, of the associated monodromy matrix, where $\varvec{v}_{{\textbf {s}}/{\textbf {u}}}$ is normalized by the magnitude of its position components [21]. Then, the perturbed state, $\varvec{x}_{{{\textbf {PO}}}} \pm d \varvec{v}_{{\textbf {s}}/{\textbf {u}}}$, is propagated backward (or forward) in time to produce a trajectory along the global stable (or unstable) half-manifold. This numerical process is repeated for the selected states along the periodic orbit to produce a discrete approximation of the desired global half-manifold over a time interval of interest.

### Numerically Correcting Transfers

Collocation is a numerical method used to implicitly integrate the differential equations of a dynamical system [1, 4, 34]. Using collocation, a solution to a dynamical system is approximated using sets of piecewise polynomials that satisfy the system dynamics at collocation nodes. In this paper, a free variable and constraint vector formulation of collocation is used to transform the trajectory design problem into a parameter design problem and robustly compute trajectories from primitive-based initial guesses. The formulation summarized in this subsection is based upon the generalized odd-degree collocation scheme with hybrid mesh refinement presented by Grebow and Pavlak [11].

#### Discretizing a Trajectory

Given an initial guess for a trajectory composed of *N* segments, the first step of collocation is to define a discrete mesh of nodes along the trajectory. The *i*-th segment is discretized into $m_{i}$ arcs and a total of $m_{i}+1$ nodes at their boundaries. The nodes generated in this discretization process are referred to as boundary nodes with each node described by its state and time. Across the *N* segments, this discretization produces a total of *m* arcs, and the time associated with the boundary node at the end of segment *i* is set equal to the time associated with the boundary node at the beginning of segment $i + 1$.

Next, an implicit integration method is selected to determine the number of nodes placed along each arc. Higher-order polynomials have successfully been used by a variety of researchers for implicit integration in nonlinear dynamical systems [4, 20, 34]. Based on previous successful applications of collocation for trajectory design in multi-body systems and the Mission Analysis, Operations, and Navigation Toolkit Environment (MONTE) Collocation tool, the degree of the polynomials is assumed to be odd and 7-th order polynomials are used in this paper [11, 24, 27]. Therefore, $n = 7$ collocation nodes are placed along each of the $m_i$ arcs within each of the *N* segments of a trajectory.

To facilitate a clear discussion of the node spacing strategy, a set of definitions and notation are established for the properties of each arc along a trajectory as well as the parameterization of the polynomials. The state and time associated with a given node are defined as $\varvec{x}_{j,k}^{i}$ and $t_{j,k}^{i}$, respectively, where *i* refers to the segment index along the trajectory, *j* refers to the arc index along the *i*-th segment, and *k* refers to the node index along the *j*-th arc in the *i*-th segment. Following this notation, the integration time along the *j*-th arc in the *i*-th segment is calculated as $\Delta t_{j}^{i} = t_{j,n}^{i} - t_{j,1}^{i}$. Furthermore, each state variable along each arc of the trajectory is approximated with a distinct 7-th order polynomial parameterized by a normalized time quantity, $\tau$, spanning from -1 to 1. The transformation from the time *t* to the normalized time $\tau$ at a state along the *j*-th arc in the *i*-th segment is defined as

3 $$\begin{aligned} \tau = 2\left( \frac{t - t_{j,1}^{i}}{\Delta t_{j}^{i}}\right) - 1 \end{aligned}$$

Then, the state vector at $t_{j,k}^{i}$ is approximated by the polynomials of the *j*-th arc in the *i*-th segment and denoted as $\varvec{p}_{j}^{i}(\tau _{k})$. Next, the normalized time derivative of the state vector $\varvec{x}_{j,k}^{i}$ is defined as

4 $$\begin{aligned} \dot{\varvec{x}}_{j,k}^{i} = \frac{\Delta t_{j}^{i}}{2} \varvec{g}(\varvec{x}_{j,k}^{i}) \end{aligned}$$

where $\varvec{g} = [\dot{x}, \dot{y}, \dot{z}, \ddot{x}, \ddot{y}, \ddot{z}]^{\text {T}}$. Finally, the normalized time derivative of the state vector $\varvec{p}_{j}^{i}(\tau _{k})$ approximated by the polynomials is denoted as $\dot{\varvec{p}}_{j}^{i}(\tau _{k})$.

**Fig. 1:**
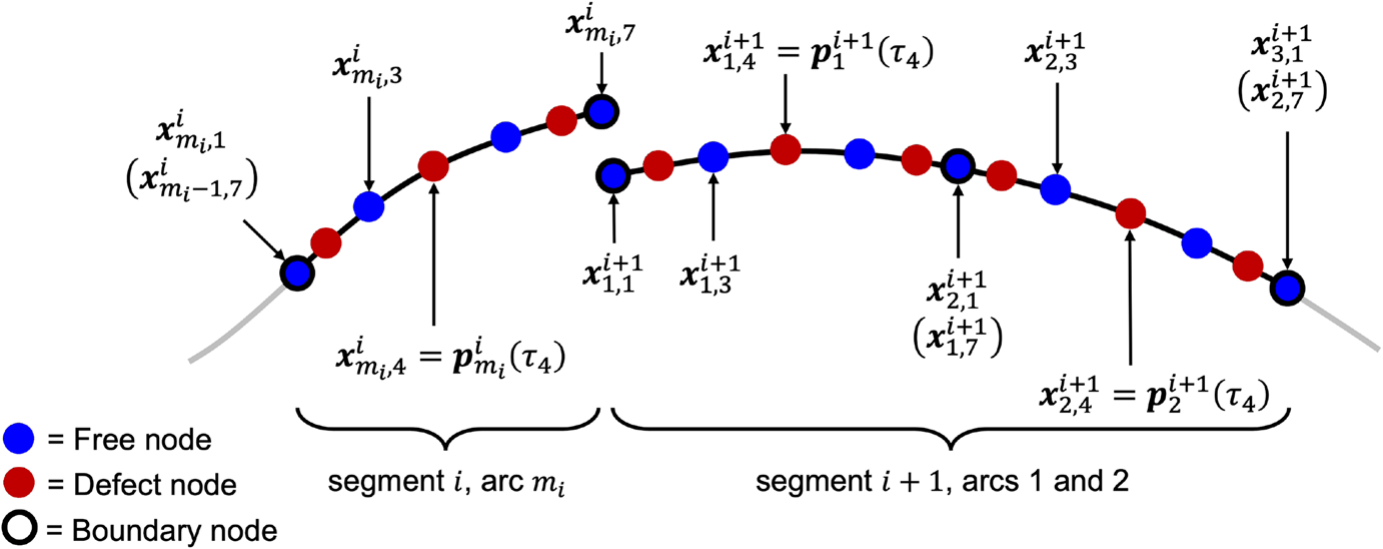
Conceptual example of collocation nodes placed along multiple arcs of segment *i* and $i+1$ using 7-th order LGL node spacing

In collocation, a node spacing strategy is used to determine the location of the collocation nodes along each arc. Legendre-Gauss-Lobatto (LGL) node spacing has successfully been used by a variety of researchers in spacecraft trajectory design [4, 11, 40]. In this method, collocation nodes are placed at the boundary nodes of each arc and at the normalized times $\tau$ equal to the roots of the derivative of the $(n-1)$-th order Legendre polynomial, ranging from - 1 to 1. Additionally, an LGL weighting term, *w*, is computed for each node. Leveraging LGL node spacing is advantageous because it simplifies the design problem by considering the boundary nodes of each arc as collocation nodes [11]. Along each arc, the odd-numbered collocation nodes are classified as free nodes and the even-numbered collocation nodes are classified as defect nodes. The free nodes are used to construct the approximating polynomials along each arc, whereas the defect nodes are used to evaluate how well the system dynamics are approximated by the polynomials. Figure 1 depicts a conceptual example with each arc containing a set of 7 nodes, including 4 free nodes (blue) and 3 defect nodes (red), as determined by the 7-th order implicit integration method using LGL node spacing. The boundary nodes (outlined in black) are considered collocation nodes and are classified as free nodes. As depicted in Fig. 1, consecutive arcs within a segment share a common free boundary node. However, the final free boundary node along segment *i* is distinct from the initial free boundary node along segment $i + 1$.

#### Correcting a Trajectory

To formulate a corrections problem that uses collocation, a free variable vector is defined using the state at each free node and the time elapsed along each of the *m* arcs of a trajectory. Mathematically, the free variable vector $\varvec{V}_{i}$ for the *i*-th segment, composed of $m_i$ arcs, is defined as

5 $$\begin{aligned} \varvec{V}_{i} = \begin{bmatrix} \begin{bmatrix} \varvec{x}_{1,1}^{i} \\ \varvec{x}_{1,3}^{i} \\ \vdots \\ \varvec{x}_{1,n - 2}^{i} \end{bmatrix}^{\text {T}} \begin{bmatrix} \varvec{x}_{2,1}^{i} \\ \varvec{x}_{2,3}^{i} \\ \vdots \\ \varvec{x}_{2,n - 2}^{i} \end{bmatrix}^{\text {T}} \cdots \begin{bmatrix} \varvec{x}_{m_{i}-1,1}^{i} \\ \varvec{x}_{m_{i}-1,3}^{i} \\ \vdots \\ \varvec{x}_{m_{i}-1,n-2}^{i} \end{bmatrix}^{\text {T}} \begin{bmatrix} \varvec{x}_{m_{i},1}^{i} \\ \varvec{x}_{m_{i},3}^{i} \\ \vdots \\ \varvec{x}_{m_{i},n}^{i} \end{bmatrix}^{\text {T}} \begin{bmatrix} \Delta t_{1}^{i} \\ \Delta t_{2}^{i} \\ \vdots \\ \Delta t_{m_i}^{i} \end{bmatrix}^{\text {T}} \end{bmatrix} \end{aligned}$$

where $n = 7$. The free variable vector for the entire trajectory then equals

6 $$\begin{aligned} \varvec{V} = \begin{bmatrix} \varvec{V}_{1}&\varvec{V}_{2}&\cdots&\varvec{V}_{N} \end{bmatrix}^{\text {T}} \end{aligned}$$

to produce a $((3n - 2)m + 6N)$-dimensional vector for *N* segments.

To compute a continuous trajectory, a set of continuity constraints must be satisfied. Continuity is automatically enforced between arcs within a segment due to the use of LGL nodes because each pair of consecutive arcs shares a common free boundary node [11]. However, continuity must be explicitly enforced between consecutive segments, resulting in the following constraint:

7 $$\begin{aligned} \varvec{F}_{{\textbf {c}}}^{i} = {\left\{ \begin{array}{ll} \varvec{x}_{1,1}^{i+1} - \varvec{x}_{m_i,n}^{i} &{}\text {if natural motion} \\ \varvec{r}_{1,1}^{i+1} - \varvec{r}_{m_i,n}^{i} &{} \text {if impulsive maneuver applied} \end{array}\right. } \end{aligned}$$

between the *i*-th and $(i+1)$-th segments.

Defect constraints must also be satisfied along each arc of the entire trajectory to enforce the system dynamics at each defect node. Each defect constraint evaluates the difference between the approximated dynamics, computed using the normalized time derivatives of the polynomials, and the actual dynamics, computed at each defect node using Eq. 4. The defect constraint vector for the *j*-th arc in the *i*-th segment is defined as

8 $$\begin{aligned} \varvec{F}_{{\textbf {d}}_j}^{i} = \begin{bmatrix} \varvec{\Delta }_{j,2}^{i} \\ \varvec{\Delta }_{j,4}^{i} \\ \vdots \\ \varvec{\Delta }_{j,n - 1}^{i} \end{bmatrix} = \begin{bmatrix} (\dot{\varvec{p}}_{j}^{i}(\tau _2) - \dot{\varvec{x}}_{j,2}^{i})w_2 \\ (\dot{\varvec{p}}_{j}^{i}(\tau _4) - \dot{\varvec{x}}_{j,4}^{i})w_4 \\ \vdots \\ (\dot{\varvec{p}}_{j}^{i}(\tau _{n - 1}) - \dot{\varvec{x}}_{j,n - 1}^{i})w_{n - 1} \end{bmatrix} \end{aligned}$$

where $n = 7$ and each $w_k$ term is the LGL weight associated with the *k*-th collocation node. Then, the defect constraint vector for the *i*-th segment is defined as $$\varvec{F}_{{\textbf {d}}}^{i} = \begin{bmatrix} \varvec{F}_{{\textbf {d}}_1}^{i^{\text {T}}}&\varvec{F}_{{\textbf {d}}_2}^{i^{\text {T}}}&\cdots&\varvec{F}_{{\textbf {d}}_{m_i}}^{i^{\text {T}}} \end{bmatrix}$$.

A constraint vector for the entire trajectory captures both the continuity and defect constraints. This constraint vector is defined as

9 $$\begin{aligned} \varvec{F}(\varvec{V}) = \begin{bmatrix} \varvec{F}_{{\textbf {c}}}^{1^{\text {T}}}&\varvec{F}_{{\textbf {c}}}^{2^{\text {T}}}&\cdots&\varvec{F}_{{\textbf {c}}}^{{N-1}^{\text {T}}}&\varvec{F}_{{\textbf {d}}}^{1}&\varvec{F}_{{\textbf {d}}}^{2}&\cdots&\varvec{F}_{{\textbf {d}}}^{N} \end{bmatrix}^{\text {T}} \end{aligned}$$

to produce a $((3n-3)m + 6(N-1) - 3P)$-dimensional vector, where *P* is the number of impulsive maneuvers applied only between consecutive pairs of segments. Using a corrections or optimization algorithm, the free variable vector may be iteratively updated from an initial guess to compute a continuous trajectory that satisfies these constraints to within a tolerance of $10^{-12}$.

The free variable and constraint vectors defined in Eqs. 6 and 9, respectively, may be modified in a straightforward manner to also include impulsive maneuvers between consecutive pairs of arcs within a given segment. For example, consider the general case of an impulsive maneuver applied between arcs *j* and $j+1$ along the *i*-th segment. In this case, $\varvec{x}_{j,n}^{i}$ is included in Eq. 6 and considered distinct from $\varvec{x}_{j+1,1}^{i}$; therefore, a position continuity constraint defined as $\varvec{F}_{{\textbf {c}}_j}^{i} = \varvec{r}_{j+1,1}^{i} - \varvec{r}_{j,n}^{i}$ must be included in Eq. 9 to account for the desired maneuver. Additional path constraints may also be incorporated into Eq. 9 depending on the design scenario.

#### Mesh Refinement

The numerical accuracy of a trajectory computed using collocation depends on its mesh of nodes. Despite the defect constraints being satisfied along each arc, the solution may not accurately approximate the system dynamics between collocation nodes, particularly in sensitive regions of the dynamical system [27]. Therefore, mesh refinement is used to improve the accuracy of the solution. In this paper, a hybrid mesh refinement algorithm is employed that follows the procedure presented by Grebow and Pavlak, which uses both analytical and numerical analysis to control the error along a trajectory [11, 27].

Once an initial trajectory is computed via corrections, Carl de Boor’s method is employed to iteratively distribute error equally between arcs along the solution [6, 11, 29]. During this process, the number of arcs in the mesh and the total flight time for the trajectory are constant, whereas the integration time along each arc (i.e., $\Delta t_{j}^{i}$) varies. At each iteration of de Boor’s method, the error along the *j*-th arc in the *i*-th segment is calculated as

10 $$\begin{aligned} e_{j}^{i} = K (\Delta t_{j}^{i})^{n+1} \xi _{j}^{i} \end{aligned}$$

where *K* is a constant that depends on the selected polynomial degree and type and $\xi _{j}^{i}$ is a scalar quantity defined based on the magnitude of the largest component of the $(n+1)$-th unnormalized time derivative estimate of $\varvec{p}_{j}^{i}(\tau )$, computed using finite differencing [6, 11, 27, 29]. Based on the error distribution of the current solution, the time at the first boundary node of the *j*-th arc in the *i*-th segment may be updated such that $t_{j,1}^{i} = t_{\text {bnd}}$ where $t_{\text {bnd}}$ satisfies the following equation:

11 $$\begin{aligned} I(t_{\text {bnd}}) = \frac{(a-1)}{m}I(t_{m_i,n}^{N}) \text {\hspace{5pt} s.t. \hspace{5pt}} I(t) = \int _{t_{1,1}^{1}}^{t} \xi (s)^{\frac{1}{n+1}} \,ds \end{aligned}$$

where *a* is the index of the associated arc along the entire solution and $\xi (s)$ is a piecewise constant function equal to $\xi _{j}^{i}$ when $s \in [t_{j,1}^{i}, t_{j,n}^{i}]$ [6, 11, 29].

Given a new time distribution of boundary nodes, the polynomials of the previous mesh are used to compute the updated state at each boundary node along the trajectory. This process equally distributes the approximated error along the entire solution between all arcs in the mesh. However, if impulsive maneuvers are applied along the trajectory, the boundary nodes are adjusted to equally distribute the cumulative error between each consecutive pair of maneuvers rather than along the entire solution. The free LGL nodes for each arc are then recomputed using the polynomials of the previous mesh between the updated boundary nodes.

The differential corrections process outlined in Sect. 2.3.2 is then used to compute a refined continuous trajectory with the updated mesh as an initial guess. This error distribution process repeats until one of the following terminal conditions is met: the maximum error difference along the current solution between any two arcs is $\le 10^{-5}$; the maximum error difference along the current solution changed by $\le$ 10% from the previous iteration; or a maximum number of iterations, selected in this paper as 5, is exceeded. The values for these termination criteria are selected empirically based on the convergence behavior of the transfers constructed in this paper.

When the error distribution step is terminated, Control with Explicit Propagation (CEP) is used to iteratively merge arcs along the mesh to reduce the size of the sparse corrections problem [11, 27]. This step of mesh refinement numerically computes the error at the end of each pair of consecutive arcs in the mesh, except when an impulsive maneuver occurs between them. For example, the state at the initial boundary node of the first arc is propagated until the time associated with the final boundary node of the second arc. Then, the error is computed between this final propagated state and the state associated with the final boundary node of the second arc. If the magnitude of the error vector is below a tolerance of $10^{-13}$, then the two arcs are merged into a single arc. In this case, the initial boundary node of the first arc and the final boundary node of the second arc serve as the initial and final boundary nodes, respectively, of the merged arc. Then, the free LGL nodes are recomputed between the updated boundary nodes using the polynomials of the previously converged mesh and the procedure is repeated for the next two consecutive arcs in the mesh. If any arcs are merged along the entire trajectory, then the updated mesh is used to compute a refined continuous trajectory by reapplying the differential corrections process outlined in Sect. 2.3.2. The merging process is repeated until no arcs are merged along the solution or a maximum number of iterations is exceeded; this threshold is selected empirically as 10 based on the convergence behavior of the transfers calculated in this paper.

After completing the merging process, CEP is also used to iteratively split arcs along the mesh by numerically computing the error at the end of each arc. For example, the state at the initial boundary node of an arc is propagated until the time associated with the final boundary node of the arc. Then, the error is computed between the final propagated state and the state at the final boundary node of the arc. If the magnitude of the error vector is above a tolerance of $10^{-12}$, then the arc is split into two separate arcs at its midpoint in terms of time. The polynomials from the previously converged mesh are used to compute the state and time at the midpoint of the arc and then the free LGL nodes are recomputed for each of the resulting arcs as previously described. If any arcs are split along the entire trajectory, then the updated mesh is used to compute a refined continuous trajectory by reapplying the differential corrections process outlined in Sect. 2.3.2. This process is repeated until no arcs are split along the solution. Similar to the merging loop, the splitting process is also terminated if a maximum of 10 iterations is exceeded.

## Primitive-Based Transfer Design Process

In this section, a primitive-based initial guess construction framework is formulated to generate trajectories in the CR3BP. This procedure consists of the following steps:

1. Construct a motion primitive library that summarizes the characteristics of arcs that exist within segments of the solution space.
2. Construct a motion primitive graph that discretely approximates a subset of the continuous solution space.
3. Search the graph for motion primitive sequences that serve as candidates for initial guesses for trajectories.
4. Construct an initial guess for each trajectory by refining each motion primitive sequence.
5. Correct each initial guess to produce a continuous trajectory with impulsive maneuvers using direct collocation and local optimization.
6. Compute additional transfers spanning segments of the design space.

This section summarizes each step of the initial guess construction process using the example of a planar transfer from an $L_1$ Lyapunov orbit to an $L_2$ Lyapunov orbit in the Earth-Moon CR3BP with impulsive maneuvers.

### Step 1: Construct a Motion Primitive Library

The first step in the initial guess construction framework is to construct a library of motion primitives along with information approximating the regions of the phase space spanned by arcs with similar properties. Although the definition of a motion primitive depends on the application, this paper uses a similar definition to Frazzoli: a set of motion primitives is a finite set of arcs that sufficiently summarize the characteristics of part of the solution space [8, 32]. In existing applications, motion primitives have been extracted using a variety of methods such as manual labeling, analytical approximations via basis functions, or clustering [8, 25, 28, 39]. Then, an initial guess for a trajectory may be coarsely constructed from an ordered sequence of motion primitives within the library [8, 22, 41]. In the absence of general analytical expressions to describe the solution space and due to the significant burden of manual labeling, this paper uses a clustering-based approach that we have previously developed to extract a set of motion primitives that summarize periodic orbits and arcs along hyperbolic invariant manifolds in the CR3BP [32]. Although this subsection offers a brief overview of this procedure, additional details appear in Smith and Bosanac 2022 [32].

To cluster a set of continuous trajectories and extract a representative set of motion primitives, each trajectory is described using a finite-dimensional feature vector that reflects its geometric, stability, and/or energy properties. For each trajectory, the geometric component of the feature vector, $\varvec{f}_{{\textbf {g}}}$, is defined using the sequence of states at the *l* apses along the trajectory measured relative to a specified reference point as

12 $$\begin{aligned} \varvec{f}_{{\textbf {g}}} = \left[ \tilde{x}_1 \hspace{10pt} \tilde{y}_1 \hspace{10pt} \tilde{z}_1 \hspace{10pt} \dot{\tilde{x}}_1 \hspace{10pt} \dot{\tilde{y}}_1 \hspace{10pt} \dot{\tilde{z}}_1 \hspace{10pt} \cdots \hspace{10pt} \tilde{x}_l \hspace{10pt} \tilde{y}_l \hspace{10pt} \tilde{z}_l \hspace{10pt} \dot{\tilde{x}}_l \hspace{10pt} \dot{\tilde{y}}_l \hspace{10pt} \dot{\tilde{z}}_l \right] \end{aligned}$$

where a tilde indicates normalization of each feature within the range $[-1,1]$; the relative position components are normalized using the global maximum distance of an apsis relative to the desired reference point among all trajectories within the dataset whereas the velocity components are normalized to produce the unit velocity vector at each apsis. For periodic orbits, the stability component of the feature vector is defined as

13 $$\begin{aligned} \varvec{f}_{{\textbf {s}}} = \left[ \tanh \left( {\frac{s_1}{2}}\right) \hspace{10pt} \tanh \left( {\frac{s_2}{2}} \right) \right] \end{aligned}$$

and the energy component, $f_{\text {e}}$, is defined as

14 $$\begin{aligned} f_{\text {e}} = {\tilde{C}}_{J} \end{aligned}$$

where the Jacobi constant is normalized using a min-max normalization based on the range of $C_J$ values for its corresponding family. Using these definitions, the feature vector summarizing a periodic orbit is defined as

15 $$\begin{aligned} \varvec{f}_{{{\textbf {PO}}}} = \left[ \varvec{f}_{{\textbf {g}}} \hspace{10pt} \varvec{f}_{{\textbf {s}}} \hspace{10pt} f_{\text {e}}\right] \end{aligned}$$

with a length of $6l_{\max }+3$ where $l_{\max }$ is the maximum number of apses along all trajectories in the set. The feature vector for an arc along a stable or unstable manifold at a single Jacobi constant is defined as

16 $$\begin{aligned} \varvec{f}_{{{\textbf {Mani}}}} = [\varvec{f}_{{\textbf {g}}} \hspace{10pt} \Delta {\tilde{t}}_1 \hspace{10pt} \cdots \hspace{10pt} \Delta {\tilde{t}}_{l-1}] \end{aligned}$$

where $\Delta {\tilde{t}}_i$ is the time between the *i*-th and $(i+1)$-th apses, normalized by the integration time of the arc, and $\varvec{f}_{{{\textbf {Mani}}}}$ possesses a length of $7l_{\max }-1$. When $l < l_{\max }$, placeholder values of zero are appended to Eqs. 12 and 16 to ensure the feature vector for each trajectory in the dataset is the same length. In this paper, these feature vectors are calculated for sets of trajectories that are comprised of either (1) periodic orbits along a single family or (2) arcs along a stable or unstable half-manifold of a periodic orbit and generated for up to a desired number of apses relative to a reference point.

Weighted Evidence Accumulation Clustering (WEAC) is applied to the feature vectors of a trajectory set to identify clusters of trajectories with similar properties [32]. The WEAC algorithm is a consensus clustering approach that generates a single clustering result from an ensemble of individual clustering results. In this paper, *k*-means and agglomerative clustering are leveraged to generate an ensemble of clustering results: for each algorithm, multiple clustering results are generated as the governing input parameters are varied [14, 32]. Then, a similarity measure is computed based on how often two trajectories are co-located in the same cluster within the ensemble of clustering results. This process is conceptually similar to gathering a consensus from a group of individuals. The final set of clusters is generated using agglomerative clustering applied to the resulting similarity matrix for the dataset. As discussed in Smith and Bosanac 2022 [32], additional cluster refinement is sometimes useful due to the potential sparsity of a trajectory dataset or the relative weighting between components of the feature vector incorrectly grouping trajectories that are similar yet geometrically distinct. In these cases, a *k*-nearest neighbor (*k*-NN) graph is constructed for each cluster using only the position components in the feature vector. Any unconnected components in the *k*-NN graph of each cluster form additional clusters. Given the final clustering results, a motion primitive is then extracted from each cluster as its medoid, i.e., the most similar member to all other members in the cluster [37]. The resulting motion primitive set is stored in the library to supply a discrete summary of the types of motion present across a set of trajectories.

The region within the phase space spanned by trajectories resembling a motion primitive supplies information that is valuable in constructing a motion primitive graph that is searched to form an initial guess. In robotics, a motion primitive is commonly defined as a type of control input or fundamental type of action a robot may take to move within its environment unless hindered by a hardware or operational constraint [8, 25, 39]. However, trajectories in the chaotic environment of the CR3BP that resemble a specific motion primitive, given the specific definition used in this paper, only exist within a particular region of the phase space. Furthermore, it may be challenging or computationally expensive to analytically or numerically describe the volumes of the phase space spanned by each cluster of similar trajectories. Thus, in the proof of concept presented in this paper, a small set of representative members from the cluster associated with each motion primitive is also stored in the motion primitive library. This set is defined as $R_\text {e}= \{\varvec{x}_{\textbf {R}}(t) \in C\}$, where $\varvec{x}_{\textbf {R}}(t)$ is one of a small number of representative trajectories that exist across cluster *C* corresponding to a specific motion primitive.

To select the representative trajectories that form the set $R_\text {e}$ for a given primitive without requiring manual labeling or a prespecified sampling scheme, clustering is used. First, the cluster *C* associated with a given motion primitive is partitioned into *k* subclusters using the *k*-means algorithm, which is computationally efficient and tends to produce evenly-sized clusters [14]. A representative trajectory is then computed as the medoid from each subcluster [32]. Appended to this set of *k* trajectories is a set of trajectories that lie at the extrema of the values of the following quantities calculated across each cluster: for periodic orbit families, the Jacobi constant; and for hyperbolic invariant manifolds, the total propagation time along an arc. Finally, if *C* contains fewer than *k* members, then all of the trajectories in *C* are labeled as representative trajectories. Although this approach used within this proof of concept admits a low complexity, a parametric approximation that directly describes the region of the phase space spanned by arcs with similar characteristics to the motion primitive is an interesting avenue for future work.

To demonstrate this step of the design process, fundamental solutions are generated to support the construction of a planar transfer from an $L_1$ Lyapunov orbit at $C_J \approx 3.1670$ to an $L_2$ Lyapunov orbit at $C_J \approx 3.1666$ in the Earth-Moon CR3BP. Both of these orbits are primitives of their associated periodic orbit families, as computed in Smith and Bosanac 2022, and are unstable [32]. Next, the planar stable and unstable half-manifolds of these $L_1$ and $L_2$ Lyapunov orbits are generated towards the Moon. Trajectories within each half-manifold are propagated until either completing up to 15 apses relative to the Moon in backward and forward time, respectively; departing through the $L_1$ or $L_2$ gateways; or impacting the Moon. These trajectories that lie along the stable or unstable manifolds of the selected periodic orbits are then sampled to produce a larger set of arcs, each spanning a shorter time interval: beginning at each perilune or apolune and propagated for up to 3 additional apses relative to the Moon, unless meeting the previous termination conditions [32].

**Fig. 2:**
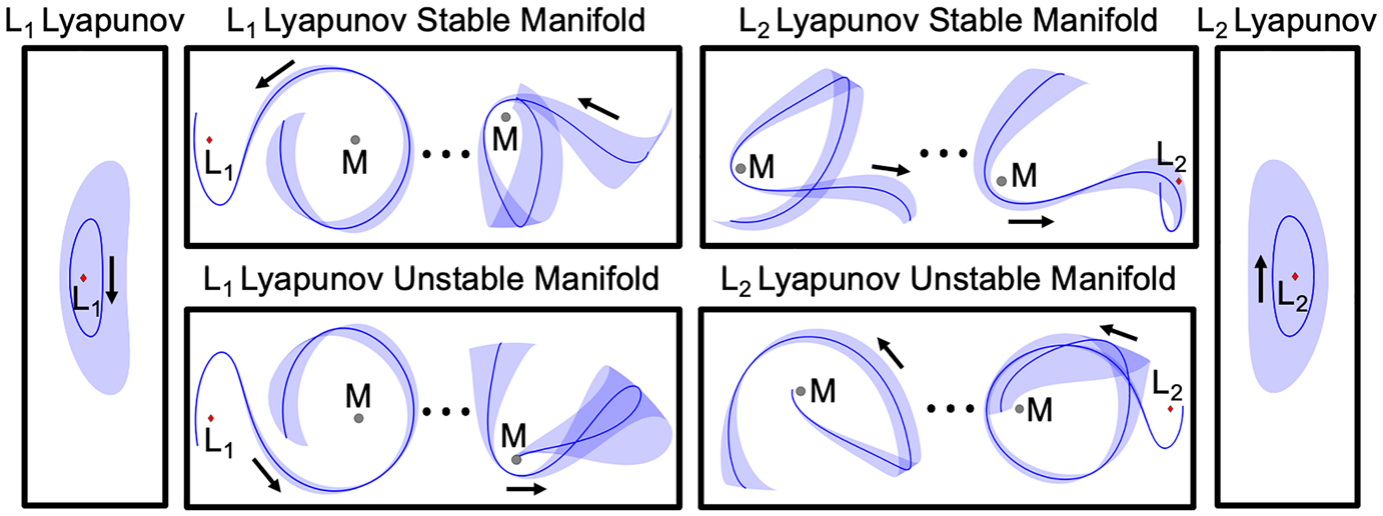
Summary of information stored in the motion primitive library for the planar $L_1$ to $L_2$ Lyapunov orbit transfer scenario in the Earth-Moon CR3BP: selected motion primitives (bold) and regions spanned by the representative trajectories of each cluster (transparent)

Motion primitives of the arcs along the stable and unstable half-manifolds of the selected $L_1$ and $L_2$ Lyapunov orbits are extracted using the procedure summarized within this section. Table 1 lists the number of primitives calculated within each set. Furthermore, Fig. 2 displays the initial $L_1$ Lyapunov orbit primitive, the target $L_2$ Lyapunov orbit primitive, and a small subset of motion primitives from their stable and unstable half-manifolds. Each primitive is denoted in bold and the region of the configuration space spanned by the associated small set of representative trajectories is depicted as a transparent surface. The entire set of primitives generated for these stable and unstable manifolds appear in Appendix A. Although the planar stable and unstable manifolds of a Lyapunov orbit are symmetric about the *x*-axis in the CR3BP, Table 1 reveals that they are not summarized by an equivalent number of motion primitives. The small difference is likely attributable to the nondeterministic nature of *k*-means clustering, which is used to generate part of the ensemble of clustering results. Specifically, *k*-means clustering is observed to sometimes produce slightly different clusters of arcs in sparsely-covered regions of the higher-dimensional space associated with the feature vectors of arcs along each of the stable and unstable manifolds. Nevertheless, these motion primitives supply a summary of the distinct geometries of arcs along each stable or unstable half-manifold. Together, they form a condensed primitive library for the example design scenario in this section. In future work, the motion primitive library may be expanded to summarize more general sets of arcs in the CR3BP and other dynamical models; this expansion will require small adjustments to the primitive extraction and graph construction processes.

**Table 1:** Motion primitives in the library for the planar transfer design scenario from an $L_1$ to $L_2$ Lyapunov orbit

Fundamental solution	Number of primitives	Approx. $C_J$
$L_1$ Lyapunov orbit	1	3.1670
$L_1$ Lyapunov orbit unstable manifold	69	3.1670
$L_1$ Lyapunov orbit stable manifold	68	3.1670
$L_2$ Lyapunov orbit	1	3.1666
$L_2$ Lyapunov orbit unstable manifold	88	3.1666
$L_2$ Lyapunov orbit stable manifold	89	3.1666

### Step 2: Construct a Motion Primitive Graph

A motion primitive graph is constructed to discretely represent a region of the continuous solution space in a multi-body system. In general, a graph is a discrete data structure composed of a set of nodes and edges that is often used to model the properties and internal relationships of a network of objects [14, 22]. When applied to motion primitive and funnel libraries, Frazzoli and Majumdar and Tedrake defined the nodes of a graph as primitives or funnels and added directed edges between nodes that may be composed in a sequence [8, 23]. Similarly, this paper defines each node in the graph as a motion primitive and its associated representative trajectories. Then, weighted, directed edges reflect the potential for pairs of primitives to be composed in a sequence to produce a nearby continuous trajectory with similar geometric properties. However, to incorporate designer expertise and reduce computational complexity, the graph construction process is composed of two steps in this paper: (1) constructing subgraphs reflecting the potential connectivity between motion primitives associated with a single type of dynamical structure and (2) constructing a modular, high-level itinerary graph to connect these subgraphs.

Formulating a motion primitive graph begins with determining the sequential composability of an ordered pair of primitives; a property that is described by Majumdar and Tedrake as their potential to produce a nearby trajectory [23]. To avoid overfitting to an incomplete approximation of the region of the phase space spanned by trajectories with similar properties to each primitive, we estimate the potential for sequential composability of two motion primitives $\varvec{x_{{{\textbf {MP}}}_i}}(t)$ and $\varvec{x_{{{\textbf {MP}}}_j}}(t)$ and, potentially, their associated sets of representative trajectories $R_{\text {e}_i}$ and $R_{\text {e}_j}$ using the following measure:

17 $$\begin{aligned} q = \alpha _{\text {pos}}\Delta r + \alpha _{\text {vel}}\Delta v \end{aligned}$$

where $\Delta r, \Delta v$ are the magnitudes of the position and velocity difference, respectively, between two primitives and, potentially, their associated sets of representative trajectories. In addition, $\alpha _{\text {pos}}$ and $\alpha _{\text {vel}}$ are selected to scale the position and velocity differences, respectively. Selecting $\alpha _{\text {vel}} \ne 0$ is useful when maneuver requirements are a high design priority. With this definition, a lower value of *q* corresponds to a higher potential for two sequentially composed motion primitives to produce a nearby continuous path when corrected with impulsive maneuvers.

To evaluate the potential for sequential composability of two motion primitives, the state difference between two trajectories is calculated. First, each trajectory is discretized: in this paper, each periodic orbit primitive is discretized into 50 states equally spaced in arclength and each manifold arc primitive is discretized into apses with respect to the Moon as well as its boundary states. Then, the state difference between two trajectories is calculated using one of the following three measures:

1. the difference between the final state of the first trajectory and the initial state of the second trajectory
2. the minimum difference between any state along the first trajectory and the initial state of the second trajectory
3. the minimum difference between the final state of the first trajectory and any state along the second trajectory

To evaluate Eq. 17, the state difference may be calculated using the primitives or both the primitives and the associated representative trajectory sets. If these representative trajectories are used, the state difference is calculated as the minimum state difference between any trajectory in the first set and any trajectory in the second set.

**Fig. 3:**
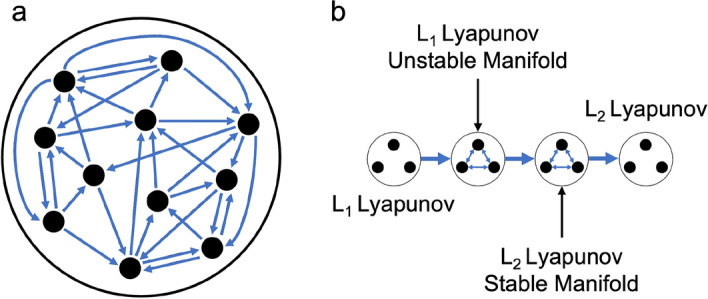
**a** Conceptual representation of a subgraph and **b** a high-level itinerary graph for the planar $L_1$ to $L_2$ Lyapunov orbit transfer scenario in the Earth-Moon CR3BP

Using the potential for sequential composability, a subgraph of each motion primitive set is independently formed. With motion primitives at each node of a subgraph, weighted and directed edges are added to the *k*-nearest neighbors of each node where $k \ge 0$ is a parameter selected by the trajectory designer. If $k = 0$, the subgraph has no internal edges and therefore motion primitives within the subgraph may not be sequentially composed. However, for $k > 0$, the neighbors for each primitive are identified using the *k* lowest values of *q* for each possible ordered primitive pair, calculated using Measure 1 between the primitives and, if desired, their associated representative trajectories. Measure 1 is used to prioritize reducing overlapping segments between pairs of primitives derived from the same dynamical structure. However, alternative measures may be used as appropriate. The edge weights are then assigned as the potential for sequential composability, *q*, for each connected pair of primitives. A conceptual representation of a subgraph is depicted in Fig. 3a where each black circle is a node in the graph and is connected to its three nearest neighbors in the set ($k = 3$). As a result, the subgraph reflects the potential for an ordered sequence of two motion primitives summarizing arcs along the same dynamical structure to be useful in the initial guess construction process.

The subgraphs are then connected according to a modular high-level itinerary graph that is constructed by the trajectory designer. In particular, the designer specifies any external connections between the subgraphs as well as the directionality of those connections. This step enables the designer to incorporate their expertise, or even lack thereof, in a scenario into the structure of the graph and, as a result, influence the possible ordering of primitives in a sequence. For each pair of connected subgraphs, each individual primitive in the source subgraph is connected to its *k*-nearest neighbors in the target subgraph via directed edges. However, there is one exception: if the target subgraph only contains the final target orbit then only the edges between the final target orbit and its *k*-nearest neighbors in the source subgraph are created. Finally, the external edge weights are assigned as the potential sequential composability between each connected pair of primitives: Measure 2 is used to compute *q* if the source primitive is a periodic orbit and the target primitive is a manifold arc but otherwise Measure 3 is used. Measure 3 prioritizes connecting the source primitive to target primitives that are closely located with its terminal state while also allowing overlapping segments between connected pairs of primitives from different subgraphs. These measures used to calculate the edge weights may also be modified by the trajectory designer as appropriate. In the resulting complete motion primitive graph, the selected value of *k* determines the degree of connectivity while also influencing the computational complexity of storing and searching the graph.

To demonstrate the presented approach, consider a high-level itinerary graph constructed using selected primitive sets from the library in Table 1 for the planar $L_1$ to $L_2$ Lyapunov orbit transfer example. A conceptual representation of this graph appears in Fig. 3b. In this figure, the arrows within the icon associated with an unstable manifold of the initial $L_1$ Lyapunov orbit indicate that the nodes of the subgraph are connected by internal edges, thereby allowing multiple primitives from the unstable manifold set to be sequentially composed in an initial guess. In contrast, the icon for the $L_1$ Lyapunov orbit denotes a subgraph with no internal edges, indicating that two primitives from this set may not be sequentially composed. The unidirectional arrows between subgraphs then indicate a desired order for composing primitives from each set. This high-level itinerary graph indicates that in this example an initial guess may only be composed of the following primitives in the specified order: one primitive from the $L_1$ Lyapunov orbit family set, one or more primitives from the unstable half-manifold of the selected $L_{1}$ Lyapunov orbit, one or more primitives from the stable half-manifold of the selected $L_{2}$ Lyapunov orbit, and one primitive from the $L_2$ Lyapunov orbit family set. If these arrows were bidirectional, then primitives from each subgraph could be composed in any order, consistent with the designer either having less insight into the transfer geometry or considering a wider variety of solution itineraries.

**Fig. 4:**
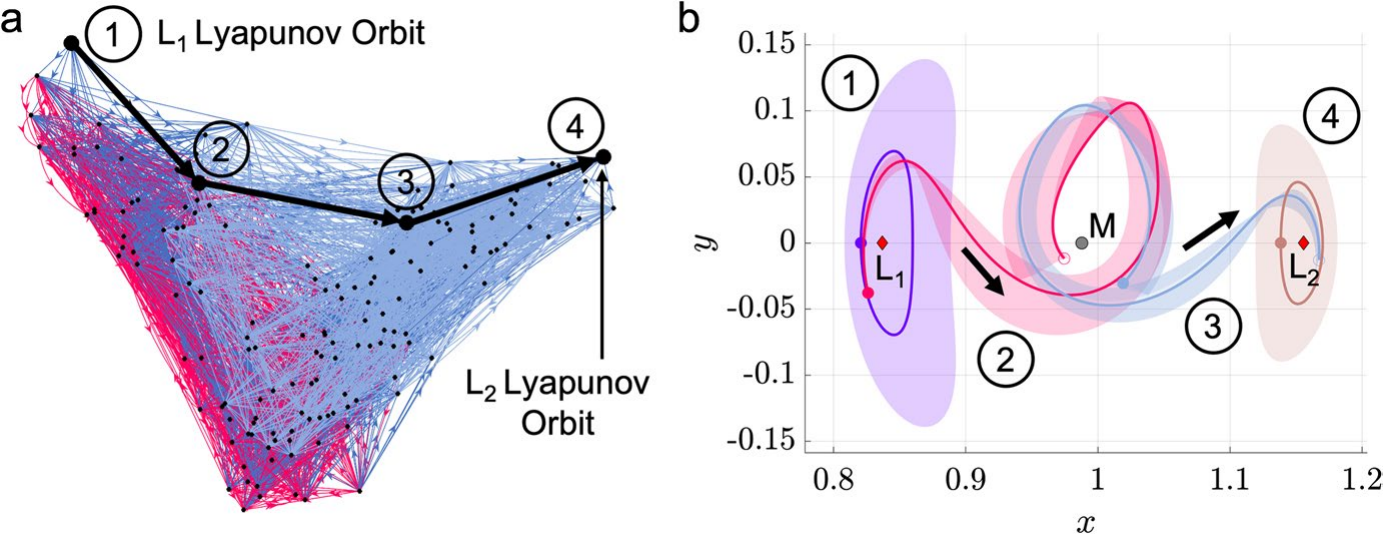
A motion primitive sequence for a planar transfer from an $L_1$ to $L_2$ Lyapunov orbit in the Earth-Moon CR3BP displayed in (a) the constructed motion primitive graph and (b) the Earth-Moon rotating frame

For the planar $L_1$ to $L_2$ Lyapunov orbit transfer example, a motion primitive graph is constructed using the high-level itinerary graph in Fig. 3b and the corresponding primitive sets from the library in Table 1. The primitives within and between each subgraph are connected with their $k = 15$ nearest neighbors using $\alpha _{\text {pos}} = 10$ and $\alpha _{\text {vel}} = 1$, which are selected empirically to emphasize position differences. These parameters may be adjusted iteratively based on the quality of the initial guesses obtained in subsequent steps and the expected maneuvering capability. Additionally, the set of representative trajectories associated with each motion primitive is incorporated into the edge weight computations. The resulting motion primitive graph is displayed in Fig. 4a: each node in the graph is depicted as a black dot, the internal edges within the $L_1$ Lyapunov unstable manifold subgraph are denoted in red, the internal edges within the $L_2$ Lyapunov stable manifold subgraph are denoted in light blue, and all external edges between nodes in different subgraphs are depicted with dark blue arrows. Although challenging for a designer to visualize, this motion primitive graph is searched to construct coarse, primitive-based initial guesses for trajectories of distinct geometries.

### Step 3: Identify Candidate Motion Primitive Sequences

A motion primitive graph is searched to produce primitive sequences that support coarsely constructing initial guesses for trajectories. In this proof of concept, the common brute-force search algorithm, depth-first search (DFS), is used to enumerate all potential paths in a motion primitive graph from an initial node to a target node with a desired length [22]; the use of alternative and more efficient search algorithms is an avenue of ongoing work. The sequence length is defined as the number of primitives in the sequence. The quality of each candidate primitive sequence in predicting a nearby continuous trajectory is then captured by the average edge weight along the path. These candidate sequences are then ranked based on their quality.

Given a ranked list of candidate primitive sequences generated from a motion primitive graph, a designer may examine all possible sequences or query the list of candidates. In this proof of concept, a straightforward filtering approach is used for rapid exploration: only the top *Q* sequences that each begin with a unique motion primitive after the initial node are examined. Of course, some transfers with unique geometries may be generated from two sequences of equal length that share a common motion primitive after the initial node. However, this approach enables a trajectory designer to systematically sift through a smaller ranked list of sufficiently distinct geometries as opposed to potentially thousands or millions of primitive sequences. An interesting avenue of ongoing work involves examining alternative approaches to extracting the best unique primitive sequences connecting the initial and target nodes.

To demonstrate this step in the context of the planar $L_1$ to $L_2$ Lyapunov orbit transfer example in the Earth-Moon CR3BP, the top-ranked sequence of four primitives with the lowest average edge weight is generated from the motion primitive graph displayed in Fig. 4a. This primitive sequence is plotted in the Earth-Moon rotating frame in Fig. 4b: each primitive is denoted in bold using a distinct color along with a transparent region generated from the associated representative trajectories. The initial (final) state of each primitive is denoted with a filled (empty) circle. This candidate sequence is not guaranteed to predict a nearby continuous trajectory with similar geometric properties. However, a trajectory designer may visually examine the primitive sequence and its average value of *q* to determine whether to perform further analysis and refinement. Although this example presents only the top-ranked sequence of four motion primitives, it supports demonstrating the coarse construction of an initial guess for a transfer using motion primitives.

### Step 4: Construct an Initial Guess from a Primitive Sequence

A motion primitive sequence is refined to improve the quality of a coarsely-constructed initial guess and facilitate a successful numerical corrections process. For instance, the primitive sequence displayed in Fig. 4b possesses state discontinuities between each consecutive pair of primitives. This sequence also exhibits a significant overlap between the second and third primitives.

The first refinement step is to morph the primitives to further reduce the state discontinuities along the initial guess. Specifically, all possible initial guesses with similar geometry to the original motion primitive sequence are constructed by using either each motion primitive or one of the associated representative trajectories. The average value of the potential sequential composability *q* along each candidate sequence of trajectory segments is computed using the selected state difference measures for each consecutive pair of primitives. The sequence of segments with the smallest average value of *q* then produces the morphed initial guess.

The second refinement step is to trim each segment in the morphed initial guess to remove any overlapping portions. The trimming process is applied only to the internal segments between the initial and final periodic orbits and is completed automatically using one of three different methods: forward, backward, or joint sequential trimming. The forward method trims each segment to start at its closest state in the phase space relative to the final state of the previous segment. Conversely, the backward method trims each segment to end at its closest state in the phase space relative to the initial state of the next segment. Finally, the joint method trims each pair of segments to begin or end at their closest states in the phase space. In each case, the difference between two individual states is evaluated using *q*.

A single trimming method is not generally applicable to all initial guesses; therefore, the trimming process that produces the best refined initial guess is selected. First, the morphed initial guess is trimmed using each of the three possible trimming methods. Then, the average value of the potential sequential composability along each trimmed sequence of segments is computed using the following measures: Measure 2 is used to measure the state difference between a periodic orbit followed by a manifold arc; Measure 1 is used to measure the state difference between each pair of manifold arcs because the interior segments have been trimmed; and Measure 3 is used to measure the state difference between a manifold arc followed by a periodic orbit. Note that Measures 2 and 3 are used here to supply flexibility in the departure or arrival locations along a periodic orbit. The trimming method that produces the lowest average value of *q* is selected to produce the refined initial guess. Finally, segments that do not exceed a specified minimum integration time are removed from the initial guess to limit numerical issues during corrections; this threshold is selected empirically as 0.01 nondimensional time units.

**Fig. 5:**
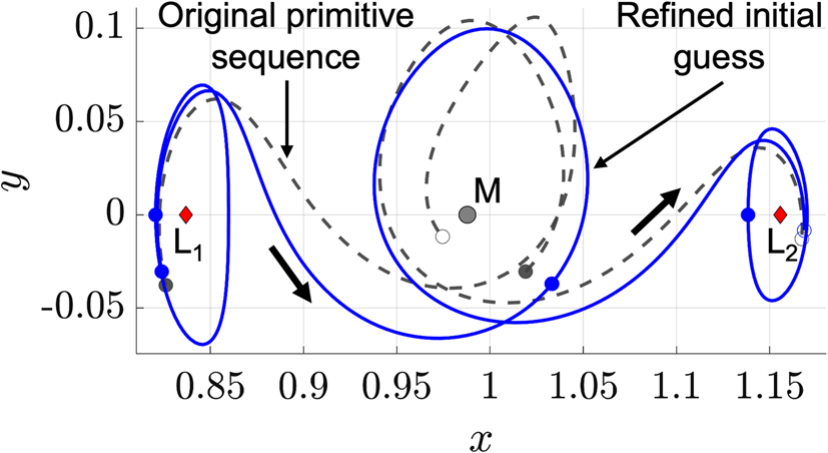
Refined primitive-based initial guess for a planar transfer from an $L_1$ to $L_2$ Lyapunov orbit in the Earth-Moon CR3BP

Using the outlined refinement process, the primitive sequence depicted in Fig. 4b is morphed and trimmed. Figure 5 displays the original primitive sequence in dashed gray and the resulting refined initial guess in blue. The overlap between the second and third arcs, as depicted in Fig. 4b, is removed using backward sequential trimming. Morphing the initial guess and then trimming the resulting segments significantly improves the quality of the initial guess in the sensitive region of the phase space near the Moon.

### Step 5: Recover a Continuous Trajectory

A continuous trajectory is computed to resemble the refined, primitive-based initial guess. In motion planning and periodic orbit computation in multi-body systems, constrained optimization methods have previously been used to compute trajectories with similar geometries as a reference path [2, 15]. However, maneuver magnitudes are also often a common concern. Thus, a multi-objective constrained optimization problem is formulated using collocation to correct a transfer between two periodic orbits with impulsive maneuvers.

First, the initial guess must be discretized. Each segment is discretized into arcs based on apses with respect to the Moon and then further discretized into an additional set of 5 arcs with equal arclength to produce an initial mesh; this discretization may be adjusted by the trajectory designer. However, if the time elapsed along the *j*-th arc in the *i*-th segment, $\Delta t_{j}^{i}$, is below a specified threshold, then it is not further discretized into smaller arcs. In this paper, this threshold is empirically selected as 0.10 nondimensional time units to avoid placing too many nodes in a short span of time, thereby facilitating better convergence behavior during corrections. Then, 7-th order polynomials and LGL nodes are used to place collocation nodes along each arc. The free nodes and $\Delta t_{j}^{i}$ along each arc of the initial guess are included in the free variable vector, as defined in Eq. 6. During corrections, the $\Delta t_{j}^{i}$ variable is constrained to remain within the bounds $[10^{-5}, 1.0]$ where the upper limit is greater than the time along any arc in the initial guess.

During corrections, the transfer is constrained to depart from any location along the desired initial orbit and arrive onto the target orbit. Thus, two additional free variables, $\Delta t_{\text {depart}}$ and $\Delta t_{\text {arrival}}$, are defined as the time elapsed from specified states along the initial and final periodic orbits. The bounds on $\Delta t_{\text {depart}}$ are set as $[-T_{\text {i}}, T_{\text {i}}]$ (initialized in $[-T_{\text {i}}/2, T_{\text {i}}/2]$) and the bounds on $\Delta t_{\text {arrival}}$ are set as $[-T_{\text {f}}, T_{\text {f}}]$ (initialized in $[-T_{\text {f}}/2, T_{\text {f}}/2]$), where $T_{\text {i}}$ and $T_{\text {f}}$ are the period of the initial and target orbit, respectively. These free variables are initialized based on the closest departure and arrival states for the initial guess.

Impulsive maneuvers are then placed at desired locations along the transfer. In this paper, maneuvers are applied at the beginning and end of the transfer, between each neighboring pair of trimmed primitives, and at apses with respect to a specified reference point. While mission requirements may constrain the placement of these maneuvers at alternative locations, this maneuver placement approach supports a proof of concept. However, if two consecutive maneuvers are placed too close together in configuration space, then one maneuver is removed: the departure/arrival maneuvers are always retained and maneuvers between consecutive trimmed primitives are prioritized above maneuvers at apses. In this paper, this threshold is empirically selected as 0.03 nondimensional distance units but may be adjusted as needed.

**Fig. 6:**
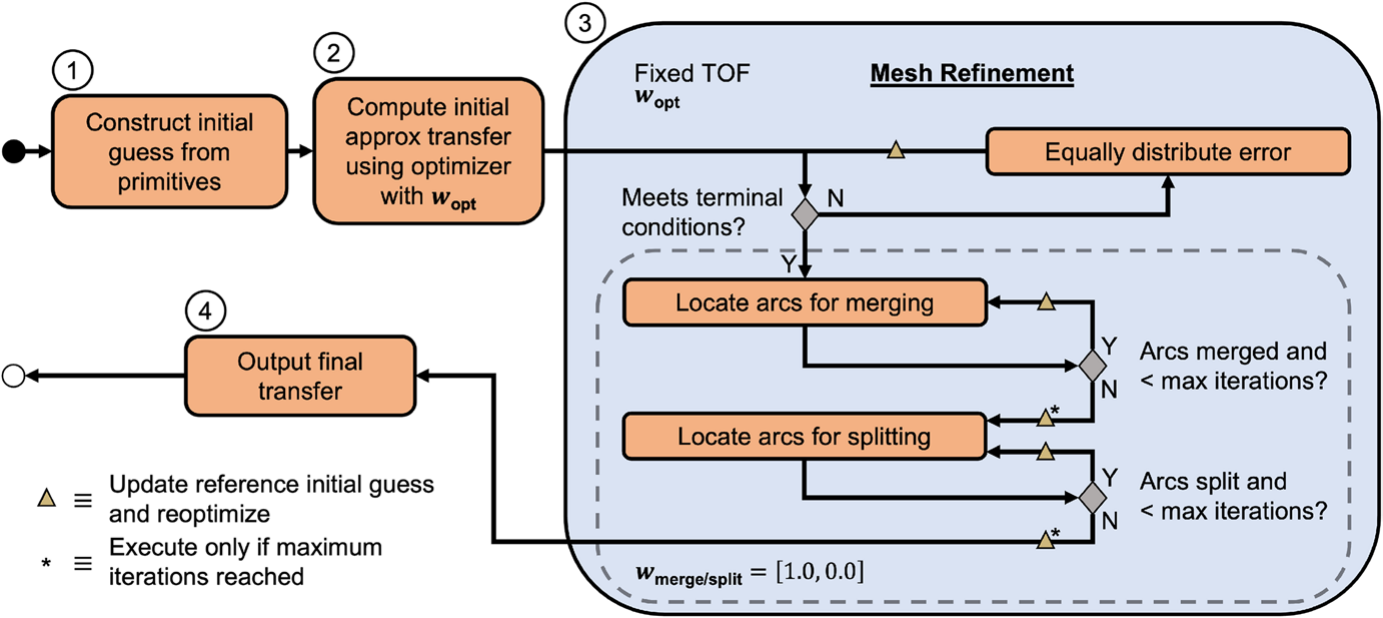
Conceptual overview of the corrections algorithm used to compute a trajectory that balances resembling a primitive-based initial guess with reducing maneuver requirements

Using these definitions, a constrained optimization problem is formulated to compute a trajectory that balances geometrically resembling a primitive-based initial guess with reducing the maneuver requirements. A summary of this procedure is depicted in Fig. 6. First, the free variable vector is defined as $\varvec{V}_{{{\textbf {trans}}}} = [\varvec{V}^\text {T}, \Delta t_{\text {depart}}, \Delta t_{\text {arrival}}]^\text {T}$ and the constraint vector is defined as $\varvec{F}_{{{\textbf {trans}}}}(\varvec{V}_{{{\textbf {trans}}}}) = [\varvec{F}(\varvec{V})^{\text {T}}, (\varvec{r}_{1,1}^{1} - \varvec{r}_{{{\textbf {depart}}}})^{\text {T}}, (\varvec{r}_{m_i,n}^{N} - \varvec{r}_{{{\textbf {arrival}}}})^{\text {T}}]^{\text {T}}$, where $\varvec{V}$ and $\varvec{F}(\varvec{V})$ are defined in Eqs. 6 and 9, respectively. Furthermore, $\varvec{r}_{{{\textbf {depart}}}}$ and $\varvec{r}_{{{\textbf {arrival}}}}$ are the departure and arrival positions along the initial and target orbit computed based on $\Delta t_{\text {depart}}$ and $\Delta t_{\text {arrival}}$, respectively. Then, an objective function is formulated as a linear combination of the difference in geometry between two trajectories and the cumulative maneuver requirements. This objective function to be minimized is defined as

18 $$\begin{aligned} J(\varvec{V}_{{{\textbf {trans}}}}) = w_{\text {geo}}((\varvec{V}_{{{\textbf {pos}}}} - \varvec{V}_{{{\textbf {IG}}}_{{{\textbf {pos}}}}})^\text {T}(\varvec{V}_{{{\textbf {pos}}}} - \varvec{V}_{{{\textbf {IG}}}_{{{\textbf {pos}}}}})) + w_{\text {man}}\left( \sum _{i=1}^{P} \Delta v_i^2\right) \end{aligned}$$

where $\varvec{V}_{{{\textbf {pos}}}}$ and $\varvec{V}_{{{\textbf {IG}}}_{{{\textbf {pos}}}}}$ reflect only the position components of the free variable vector at the current iteration and the free variable vector of the initial guess, respectively; $\varvec{w}_{{{\textbf {opt}}}} = [w_{\text {geo}}, w_{\text {man}}]$ are the relative weights of the geometric difference and maneuver requirement terms, respectively; $\Delta v_i$ is the magnitude of the *i*-th impulsive maneuver; and *P* is the total number of maneuvers. Given an initial guess, the open source Interior Point OPTimizer (IPOPT) software library equipped with the MA97 linear solver from the Harwell Subroutine Library (HSL) is used to solve the constrained optimization problem with the selected values of $w_{\text {geo}}$ and $w_{\text {man}}$ within a maximum of 1000 iterations [18, 38].

The mesh associated with the solution to the constrained optimization problem is then refined to ensure the trajectory approximated by a sequence of polynomials meets a desired level of accuracy. The mesh refinement process, depicted in Block 3 of Fig. 6, is implemented using the approach outlined in Sect. 2.3.3 while holding the time-of-flight (TOF) of the trajectory constant. After each refinement step, the updated mesh supplies an initial guess for a trajectory that is corrected via optimization as indicated with a gold triangle in Fig. 6. This optimization step during the merging and splitting phases of refinement uses $w_{\text {geo}} = 1.0$ and $w_{\text {man}} = 0.0$ to prioritize preserving the geometry of the refined solution. Each refinement step continues until the terminal conditions outlined in Sect. 2.3.3. Then, the final output is a continuous trajectory, to within a desired accuracy, that balances minimizing maneuver requirements and retaining the geometry of the initial guess.

**Fig. 7:**
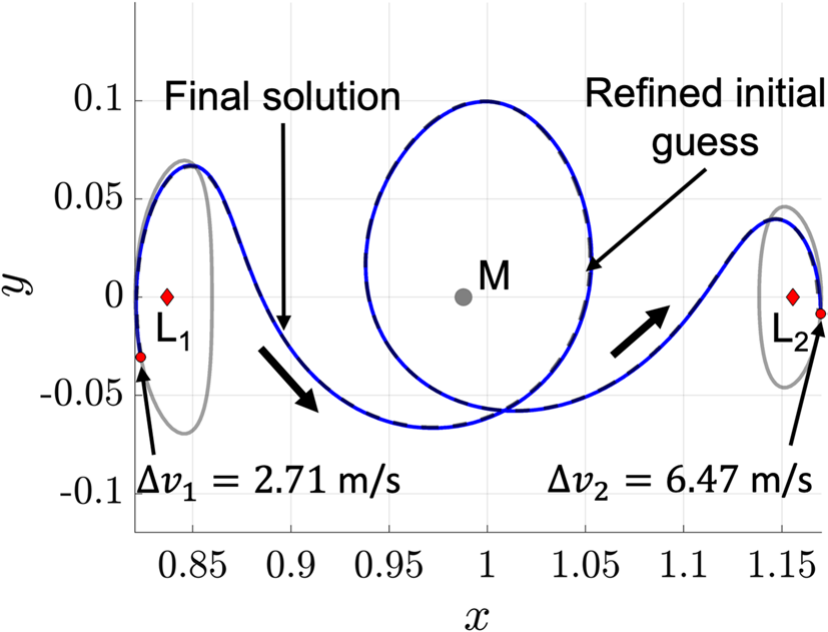
Continuous 22.34 day planar transfer from an $L_1$ Lyapunov orbit to an $L_2$ Lyapunov orbit in the Earth-Moon CR3BP, computed from a primitive-based initial guess

The numerical corrections procedure summarized in Fig. 6 is applied to the initial guess displayed in Fig. 5 for the $L_1$ to $L_2$ Lyapunov orbit transfer example. The objective function weights in Eq. 18 are selected as $\varvec{w}_{{{\textbf {opt}}}} = [0.9,0.1]$ to prioritize maintaining the transfer geometry of the initial guess while computing a more maneuver-efficient solution. Of course, these weights may be adjusted to prioritize a different balance of these two objectives. Following optimization, the resulting continuous trajectory is displayed in Fig. 7 with the refined initial guess displayed in dashed gray, the initial and target periodic orbits displayed in solid gray, and the final continuous solution displayed in solid blue. The corrected transfer includes a departure maneuver of $2.71 \hspace{4pt} \text {m/s}$, an arrival maneuver of $6.47 \hspace{4pt} \text {m/s}$, a total $\Delta v$ of $9.18 \hspace{4pt} \text {m/s}$, and a TOF between the initial and final periodic orbits that is equal to 22.34 days. This trajectory closely resembles the refined initial guess due to the objective function formulation, the selected values of the coefficients $w_{\text {geo}}$ and $w_{\text {man}}$, and the quality of the initial guess. Despite the foundational nature of this example, it demonstrates the procedure for using motion primitives to coarsely construct an initial guess with a desired transfer geometry and generate a nearby continuous trajectory.

### Step 6: Explore the Transfer Design Space

**Fig. 8:**
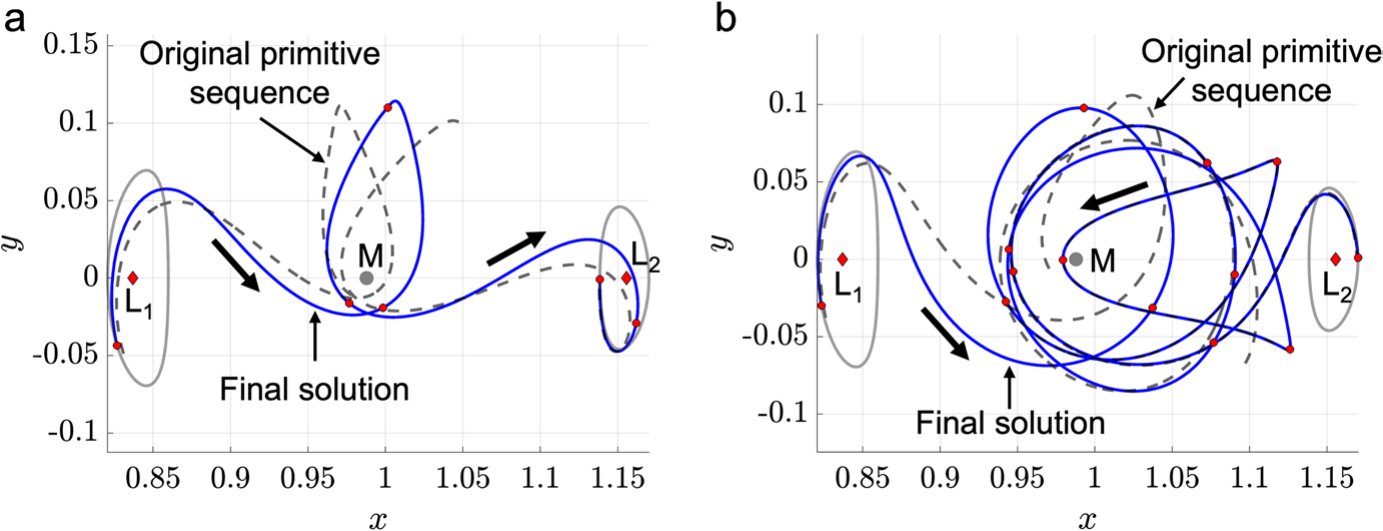
Additional transfers from an $L_1$ Lyapunov orbit to an $L_2$ Lyapunov orbit in the Earth-Moon CR3BP, computed from distinct primitive sequences

The motion primitive graph is searched to produce a variety of motion primitive sequences that could lead to transfers of distinct geometries. Using the search method outlined in Sect. 3.3, these sequences may possess either distinct lengths or correspond to the top *Q* ranked motion primitive sequences of the same length. Then, each unique sequence of motion primitives produced during this search is used to construct an initial guess. Figure 8 displays an example of two geometrically distinct planar transfers computed from distinct primitive sequences for the $L_1$ to $L_2$ Lyapunov orbit transfer design scenario explored throughout this section. The single revolution transfer displayed in Fig. 8a is constructed from an alternative four-primitive sequence to the transfer presented in Fig. 7. On the other hand, the multi-revolution transfer displayed in Fig. 8b is constructed from a six-primitive sequence. Repeating this procedure for each unique sequence of motion primitives produces a set of continuous transfers with various geometries.

Natural parameter continuation is also used to compute a set of trajectories with gradually varying paths, flight times, and maneuver requirements for each unique primitive sequence. These transfers are generated by varying the weights $w_{\text {geo}}$ and $w_{\text {man}}$ used in the multi-objective optimization process described in Sect. 3.5. In this paper, these weights are linearly varied from $\varvec{w}_{{{\textbf {opt}}}} = \varvec{w_1} = [0.9, 0.1]$ to $\varvec{w}_{{{\textbf {opt}}}} = \varvec{w_2} = [0.1, 0.9]$ in steps of 0.05. As these weights are varied, the corrected solution from the previous iteration serves as the initial guess for the current iteration; however, after the first solution is computed at $\varvec{w}_{{{\textbf {opt}}}} = \varvec{w_1}$, the error distribution step is omitted to reduce computational time. To prevent significant changes in the solution during each optimization step, the transfer TOF is also constrained to not exceed an increase of 5% from the last computed solution; this threshold may be adjusted as desired. Finally, the continuation process is terminated early if the optimizer does not converge on a solution at any iteration.

**Fig. 9:**
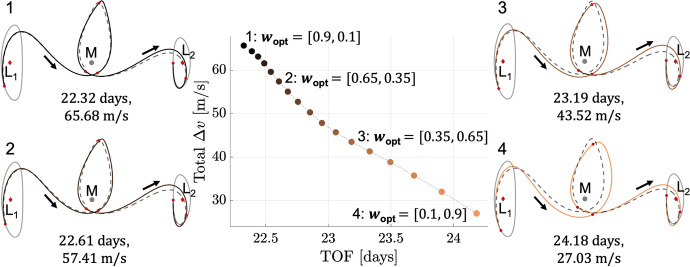
TOF and maneuver requirements of transfers in the local vicinity of the point solution displayed in Fig. 8a, computed using the continuation procedure that varies the weights of the multi-objective optimization problem

The natural parameter continuation process is applied to the transfer displayed in Fig. 8a. The center of Fig. 9 presents a summary of the flight time and total $\Delta v$ of each transfer computed with distinct values of the weights $w_{\text {geo}}$ and $w_{\text {man}}$. Although trajectories are not directly optimized with respect to flight time or total $\Delta v$ in this paper, these are common parameters of interest in the trajectory design process. For clarity, the characteristics of each transfer are indicated by a marker that varies from black when $\varvec{w}_{{{\textbf {opt}}}} = \varvec{w_1}$ to copper when $\varvec{w}_{{{\textbf {opt}}}} = \varvec{w_2}$. For selected values of these weights, annotated and numbered in the center of Fig. 9, the associated transfers are plotted at the boundaries of the figure. These results demonstrate that slight changes in the path and TOF occur as additional transfers are generated with lower maneuver requirements in the local vicinity of the solution in Fig. 8a.

Following refinement and natural parameter continuation, the complete set of generated transfers are grouped by their geometry using a *k*-NN graph. The geometric difference between two transfers, *A* and *B*, is assessed in this paper using a modified Hausdorff distance, $d_{H}(A,B)$, that is calculated as

19 $$\begin{aligned} d_{H}(A,B) = \max \left( \frac{\sum _{i=1}^{N_A} \min \limits _{j=1,...,N_B} \Vert \varvec{r}_{\varvec{A}_i} - \varvec{r}_{\varvec{B}_j}\Vert }{N_A},\frac{\sum _{i=1}^{N_B} \min \limits _{j=1,...,N_A} \Vert \varvec{r}_{\varvec{B}_i} - \varvec{r}_{\varvec{A}_j}\Vert }{N_B}\right) \end{aligned}$$

where $N_A$ ($N_B$) is the number of states sampled along trajectory *A* (*B*), $\varvec{r}_{\varvec{A}_i}$ ($\varvec{r}_{\varvec{B}_i}$) is the *i*-th position vector sampled along trajectory *A* (*B*) and measured relative to the Moon, and $\Vert \cdot \Vert$ is the $l^2$-norm [7]. Each transfer is sampled using the initial state of each arc of the mesh generated during corrections and the final state of the transfer. By using a modified Hausdorff distance, each transfer may be sampled with a distinct number of nodes. Then, a *k*-NN graph is constructed by connecting each transfer to its *k* nearest neighbors, assessed using $d_{H}(A,B)$, via edges. However, if two transfers do not mutually consider each other a *k*-nearest neighbor, their edge is removed; this step assists with identifying a single transfer with a unique geometry. Each group of geometrically similar transfers is then identified as each connected component in the *k*-NN graph. Finally, manual inspection is used to separate any incorrectly grouped transfers with distinct numbers of revolutions around the Moon.

### Summary of Governing Parameters

There are several governing parameters that a designer must specify across the entire primitive-based trajectory design framework. Table 2 summarizes these parameters and decisions. Initially, the parameters that govern the motion primitive library and graph construction processes may be selected to reflect the expertise of the designer, or even lack thereof, in a specific scenario. If the generated initial guesses exhibit insufficient geometric diversity or quality, the designer may iterate on the decisions governing the arcs summarized in the motion primitive library, the primitive extraction process, and/or the structure and connectivity of the modular high-level itinerary graph. The designer may also update the parameters governing the primitive sequences selected for further analysis and the corrections process, impacting the required analytical and computational workload. They may also adjust these parameters as they gain intuition into the success of correcting coarsely-constructed primitive-based initial guesses in each new problem. Although these parameters are currently user-selected, implementing guided or automated approaches for selecting and updating some of these parameters is an interesting avenue of future work.

**Table 2:** Governing parameters and decisions specified by a designer throughout the primitive-based initial guess construction framework

Governing parameter or decision	Step	Influence on trajectory design process
Arcs to summarize via motion primitives	1	Selection constrains region of the solution space summarized by motion primitive library; may be selected as families of fundamental solutions or general arcs
Feature vector used to describe each arc	1	Influences assessment of similarity between generated arcs during motion primitive extraction
Clustering parameters	1	Governs final groupings of similar arcs and, therefore, the number of motion primitives
Number of representative trajectories stored with each primitive	1	Influences quality of initial guess and computational time during the motion primitive graph construction and morphing processes
High-level itinerary graph structure	2	Constrains connectivity of motion primitives and, therefore, possible sequences of arcs used to form an initial guess for a trajectory. Also influences computational time
Measure used to assess $\Delta r$, $\Delta v$ in Eq. 17	2	Influences whether and how two segments with overlapping arcs may appear in a primitive sequence
$\alpha _{\text {pos}}$, $\alpha _{\text {vel}}$ weights in Eq. 17	2	Influences relative importance of position and velocity discontinuities when constructing and ranking motion primitive sequences
*k*-nearest neighbors for edge construction in motion primitive graph	2	Larger values of *k* may produce more candidate primitive sequences but also increases the computational complexity of generating and storing the graph
*Q* top-ranked primitive sequences	3	Influences number of transfers analyzed and corrected
Minimum integration time of a segment during trimming	4	Limits numerical issues during corrections
Minimum integration time of an arc during initial discretization	5	Limits initially placing too many nodes over brief time interval and, therefore, limits numerical issues during corrections
Bounds on $\Delta t_{j}^{i}$ during corrections	5	Facilitates better convergence behavior because each arc corresponds to a single integration step in the collocation method
Differential corrections and mesh refinement tolerances	5	Select based on acceptable numerical constraint violations and state discontinuity errors along a trajectory
*k*-NN graph to group geometrically similar transfers	6	Iteratively adjust *k* by visually inspecting the resulting transfer groups

## Results: Primitive-Based Transfer Design Space Exploration

The primitive-based initial guess construction framework enables the generation of trajectories with distinct geometries. In this section, this framework is used to generate transfers in the Earth-Moon CR3BP with impulsive maneuvers. Specifically, a subset of the design space is explored for planar transfers between selected $L_1$ and $L_2$ Lyapunov orbits as well as spatial transfers between selected $L_1$ and $L_2$ northern halo orbits.

### Planar Transfers from an $L_1$ to $L_2$ Lyapunov Orbit

**Fig. 10:**
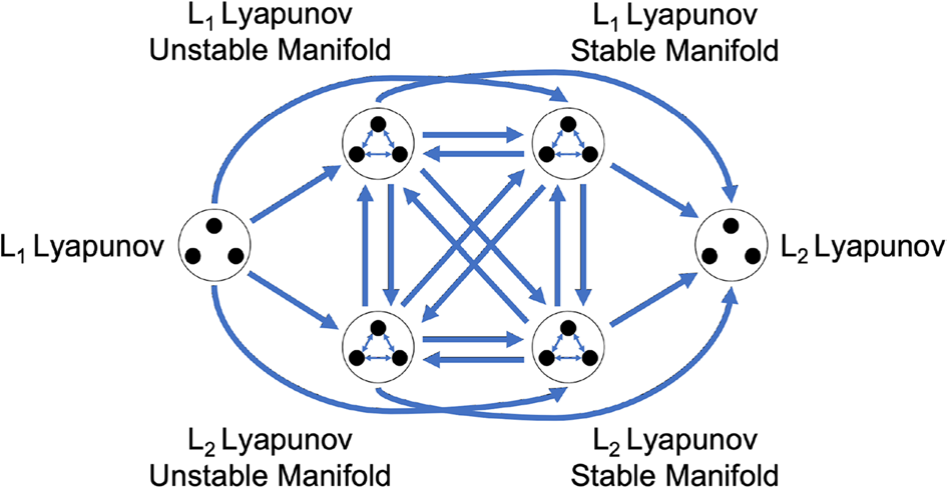
High-level itinerary graph for a planar $L_1$ to $L_2$ Lyapunov orbit transfer design scenario in the Earth-Moon CR3BP

Planar transfers are constructed from an $L_1$ Lyapunov orbit at $C_J \approx 3.1670$ to an $L_2$ Lyapunov orbit at $C_J \approx 3.1666$ in the Earth-Moon CR3BP with impulsive maneuvers. These transfers are computed using a motion primitive graph that includes primitives extracted from both the stable and unstable manifolds of the initial and final orbits, as listed in Table 1. Although existing Poincaré mapping strategies are often used to effectively visualize these types of stable and unstable manifolds, using motion primitives in this foundational planar transfer design scenario supports (1) verifying that complex initial guesses can be constructed and (2) demonstrating the recovery of a wide variety of transfer geometries. Furthermore, this approach may reduce the burden of analyzing arcs along manifolds that become increasingly complex with additional revolutions around the Moon. The associated high-level itinerary graph is depicted in Fig. 10, allowing motion primitives that summarize arcs along each manifold to be composed in any order. Accordingly, this graph expands both the array of primitives and their potential sequences compared to the graph in Fig. 3b previously used for demonstration purposes in Sect. 3. The graph used in this subsection may reflect that the trajectory designer possesses limited insight into a desired itinerary or their interest in exploring a wider region of the solution space. To construct the motion primitive graph, the following configuration parameters are used: $k = 15$, $\alpha _{\text {pos}} = 10$, and $\alpha _{\text {vel}} = 1$. Additionally, the set of representative trajectories associated with each motion primitive is incorporated into the edge weight computations and the average edge weight is used to evaluate the quality of each primitive sequence.

Various initial guesses are constructed by searching the motion primitive graph in Fig. 10 to produce unique sequences of four, five, and six motion primitives. There are 665, 45,202, and 2,681,481 primitive sequences from the initial node to the target node in the graph consisting of four, five, and six primitives, respectively; of course, not all primitives sequences necessarily predict the existence of a nearby continuous and maneuver-enabled trajectory. However, using the filtering process presented in Sect. 3.3, the $Q = 15$ top-ranked sequences that begin with a unique primitive are examined for each path length. The result is 45 primitive sequences that are each refined to produce an initial guess for a transfer. The computation time required to search for and construct these 45 primitive-based initial guesses is on the order of $10^0$ minutes using an iMac with a 3GHz 6-Core Intel Core i5 processor; a majority of this computation time is devoted to searching for the sequences of six motion primitives. The time complexity of searching the motion primitive graph using a DFS and refining each top-ranked motion primitive sequence increases significantly as the path length increases in this combinatorial problem. However, the use of alternative search algorithms is an avenue of ongoing work.

Each transfer is corrected with several unconstrained impulsive maneuvers. Specifically, each initial guess uses the maneuver placement scheme defined in Sect. 3.5 where apses are computed with respect to the Moon. Then, all 45 initial guesses are successfully corrected using $\varvec{w}_{{{\textbf {opt}}}} = [0.9, 0.1]$ to produce nearby continuous, maneuver-enabled, and planar transfers from the desired initial $L_1$ Lyapunov orbit to the target $L_2$ Lyapunov orbit.

**Fig. 11:**
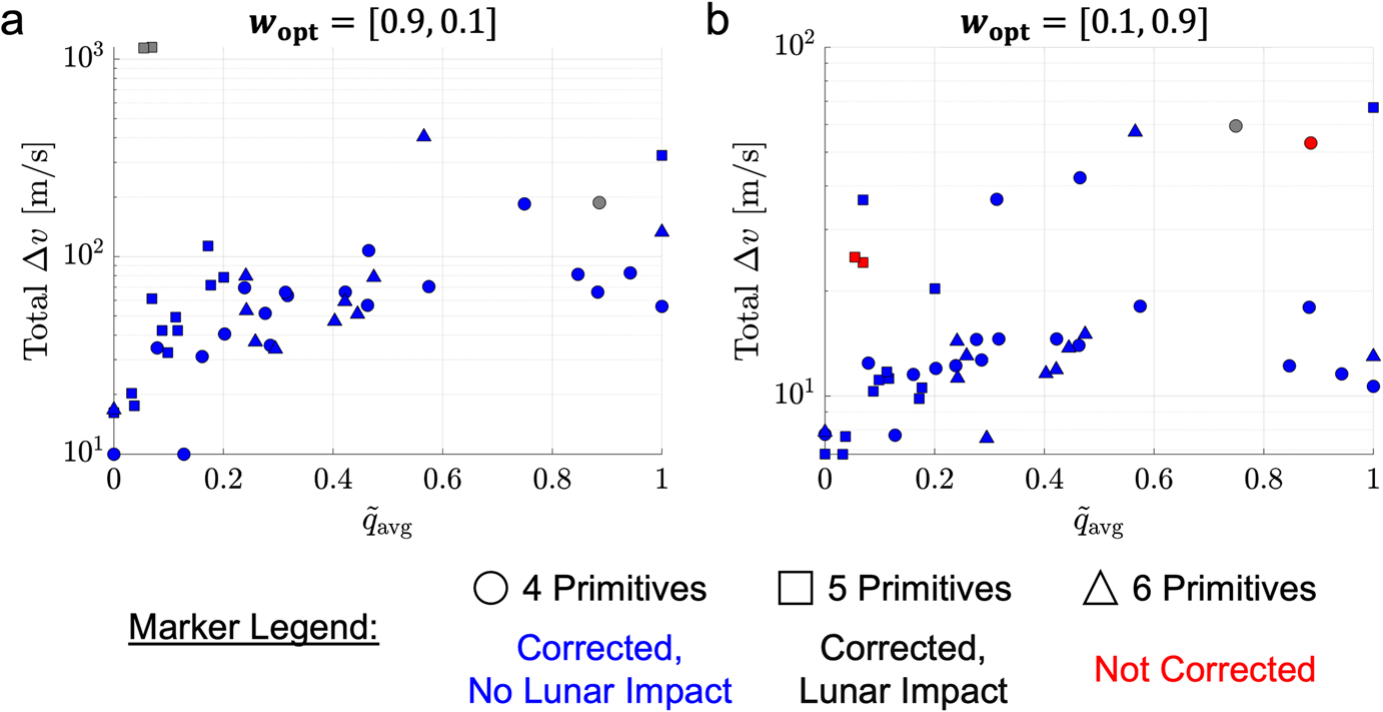
Total $\Delta v$ of planar transfers computed from an $L_1$ Lyapunov orbit to an $L_2$ Lyapunov orbit in the Earth-Moon CR3BP as a function of ${\tilde{q}}_{\text {avg}}$ using (a) $\varvec{w}_{{{\textbf {opt}}}} = [0.9, 0.1]$ and (b) $\varvec{w}_{{{\textbf {opt}}}} = [0.1, 0.9]$

An initial summary of the transfers that solve the multi-objective optimization problem with $\varvec{w}_{{{\textbf {opt}}}} = [0.9, 0.1]$ is presented using the cumulative maneuver requirements for each transfer and the sequential composability of the associated refined initial guesses. In Fig. 11a, the total $\Delta v$ of each transfer is displayed on the vertical axis using a $\log _{10}$ scale and the horizontal axis displays the normalized average potential for sequential composability, ${\tilde{q}}_{\text {avg}}$, of the refined initial guess for each transfer; a min-max normalization scheme is used to normalize $q_{\text {avg}}$ between 0 and 1 for each path length. In Fig. 11a, the properties of corrected transfers that do not impact a spherical approximation of the Moon are indicated with blue markers, whereas gray markers correspond to continuous transfers that impact the Moon. Although there is no explicit altitude constraint during corrections, only three of the corrected transfers impact the Moon. Furthermore, red markers, where applicable, correspond to discontinuous trajectories that are not successfully corrected; their values of total $\Delta v$ are estimated using the free variable vector at the final iteration of the optimization algorithm. Finally, as denoted in Fig. 11, the shape of each marker indicates the number of sequentially composed motion primitives used to compute the associated transfer. Across the set of 45 transfers, Fig. 11a reveals a gradual increase in total $\Delta v$ requirements, from $9.96 \hspace{4pt} \text {m/s}$ to $1150.83 \hspace{4pt} \text {m/s}$, with increasing values of ${\tilde{q}}_{\text {avg}}$. A wide range of $\Delta v$ requirements is expected given the variability in the quality of each initial guess, the emphasis placed on recovering transfers that resemble their respective initial guesses, and the use of unconstrained impulsive maneuvers.

Continuation is used to compute additional transfers that prioritize minimizing maneuver requirements more heavily than resembling the initial guess. Specifically, each of the 45 corrected transfers displayed in Fig. 11a forms an initial guess for the natural parameter continuation process discussed in Sect. 3.6. The relative weights are gradually varied from $\varvec{w_1} = [0.9, 0.1]$ to $\varvec{w_2} = [0.1, 0.9]$ and only 42 transfers are successfully corrected to solve the optimization problem with $\varvec{w_2}$; three transfers could not be computed using these scalar weights, likely due to numerical sensitivities near the Moon. In Fig. 11b, these transfers are summarized using the same configuration as Fig. 11a. Using this continuation-based approach, the corrected transfers possess cumulative maneuver requirements between $6.81 \hspace{4pt} \text {m/s}$ and $67.18 \hspace{4pt} \text {m/s}$, which are significantly lower than the original transfers summarized in Fig. 11a.

**Fig. 12:**
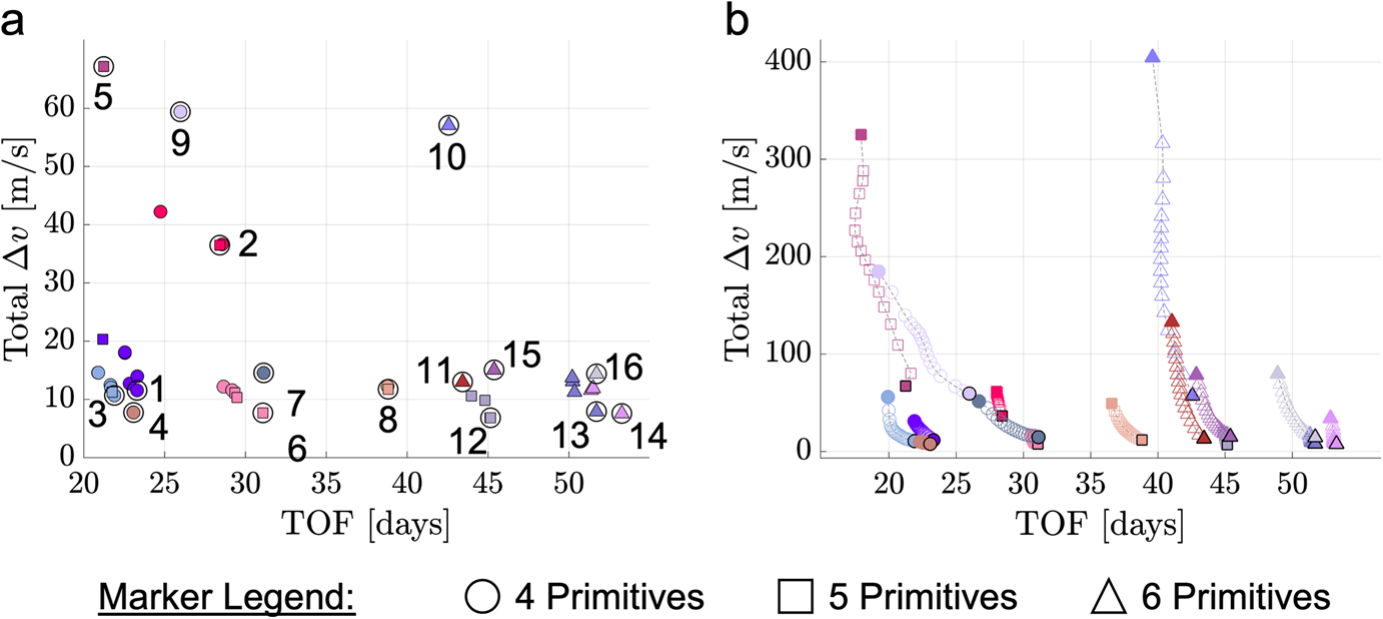
Total $\Delta v$ and TOF of transfers computed from an $L_1$ Lyapunov orbit to an $L_2$ Lyapunov orbit in the Earth-Moon CR3BP using $\varvec{w}_{{{\textbf {opt}}}} = [0.1, 0.9]$, where geometrically similar transfers are denoted in the same color and the minimum $\Delta v$ solution for each transfer geometry is highlighted

To effectively examine a set of transfers that exist within a subset of the design space, the transfers summarized in Fig. 11b are grouped by their geometry using the process described in Sect. 3.6. In this example, $k = 3$ is selected empirically to construct the *k*-NN graph. Figure 12a displays the resulting total $\Delta v$ of each transfer with respect to its TOF, with each group of geometrically similar transfers indicated by distinctly colored markers. The transfer with the minimum $\Delta v$ cost from each group is highlighted with a black circle and numbered. A total of 16 distinct types of transfers are extracted from the set of 42 planar, maneuver-enabled transfers that are corrected using $\varvec{w_2}$ to connect the selected $L_1$ Lyapunov orbit to the target $L_2$ Lyapunov orbit in the Earth-Moon CR3BP. As displayed in Fig. 12a, the corrected transfers possess flight times ranging from 20.90 days to 53.28 days and maneuver requirements from $6.81 \hspace{4pt} \text {m/s}$ to $67.18 \hspace{4pt} \text {m/s}$.

The continuation process for each transfer generally results in significant reductions in total maneuver cost coupled with increases in TOF. This information is evident in Fig. 12b, which displays the evolution of the characteristics of the minimum $\Delta v$ solution from each group computed with $\varvec{w_2}$ during the continuation process. In this figure, the solutions computed using $\varvec{w_2}$ are indicated by a filled marker with a black edge while the associated solutions computed using $\varvec{w_1}$ are indicated by only a filled marker with no edge color. These results indicate that motion primitives support the coarse design of a set of initial guesses for trajectories with distinct geometries that may also be refined to possess various flight times and maneuver requirements.

**Fig. 13:**
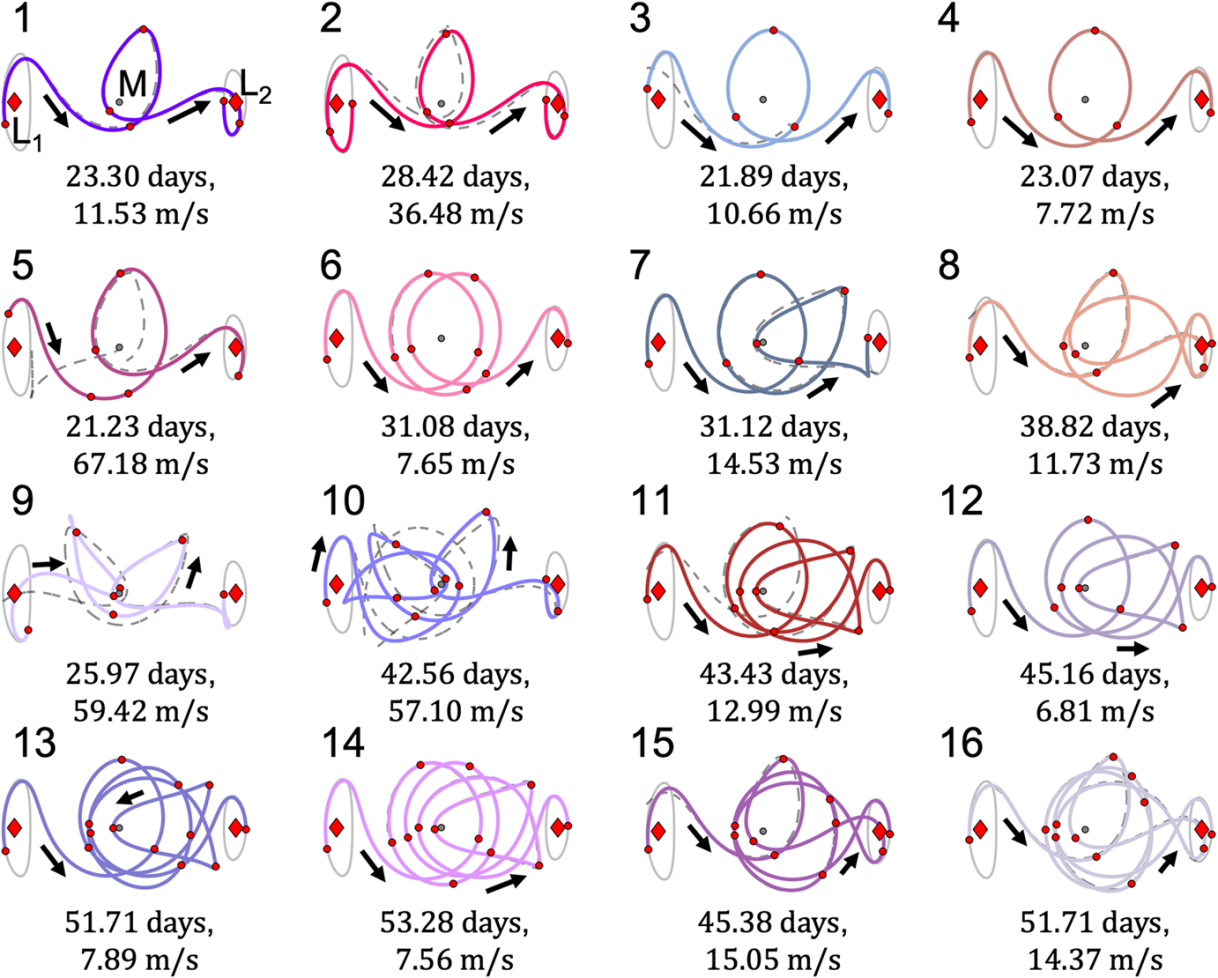
Transfers with distinct geometries computed from primitive-based initial guesses between an $L_1$ and $L_2$ Lyapunov orbit displayed in the *xy*-plane of the rotating frame in the Earth-Moon CR3BP

To visualize the geometric variations across the recovered set of transfers, the minimum $\Delta v$ transfer from each of the 16 groups is plotted in the configuration space. These transfers are displayed in Fig. 13 in the *xy*-plane of the Earth-Moon rotating frame using the same colors and numbering scheme as in Fig. 12a. In each figure, the Moon is displayed as a gray circle while $L_1$ and $L_2$ are depicted with red diamonds. The refined primitive-based initial guess for each transfer is displayed in dashed gray, the initial and target orbits are displayed in solid gray, each impulsive maneuver is located with a red circle, and black arrows indicate the direction of motion. Below each transfer is the associated flight time and total $\Delta v$. These transfers recovered using the presented motion primitive approach to trajectory design exhibit a variety of geometries while also revealing some avenues for future work.

Most of the minimum $\Delta v$ transfers that solve the multi-objective optimization problem using $\varvec{w_2}$ closely resemble their refined primitive-based initial guess. In Fig. 13, the initial guess (dashed gray) lies close to the continuous transfer (solid color) in most cases, indicating the utility of coarsely designing transfers with specific geometries using motion primitives when ${q}_{\text {avg}}$ is sufficiently low. Noticeable deviations between the corrected transfer and initial guess are, however, evident in Transfers 5, 9, and 10, which are derived from motion primitive sequences with larger discontinuities, i.e., larger values of ${q}_{\text {avg}}$. Furthermore, prioritizing maneuver requirements more heavily than resembling the initial guess can lead to more significant changes in geometry when an initial guess possesses larger discontinuities. Finally, Transfer 4 uses the same primitives as the example presented in Sect. 3. This result demonstrates the capacity to recover similar, straightforward solutions when limited a priori knowledge is incorporated into the graph construction process.

The *k*-NN graph approach for grouping transfers based on their geometry may separate transfers that could potentially belong to the same group or place transfers in a single group that could be separated. In some cases, transfer groups that are identified as possessing distinct geometries only vary in their departure or arrival locations on the initial or target orbits. For instance, Transfers 3 and 4 are constructed from unique primitive sequences and have slightly different departure locations along the initial orbit but share a similar geometry, flight time, and total $\Delta v$; a similar observation holds for Transfers 11 and 12. These transfers could potentially be considered to belong within the same transfer group but are located in disconnected components of the *k*-NN graph. Alternative values for *k*, incorporation of the transfers constructed during natural parameter continuation, or modification of the grouping process may be avenues for future work to address this observation.

The solutions presented in Fig. 13 reveal that transfers constructed from sequences of additional primitives generally exhibit complex geometries but often contain some common elements with the transfers constructed from fewer primitives. For example, Transfers 15 and 16 initially exhibit a similar geometry to Transfer 8 but perform additional revolutions around the Moon before approaching the target $L_2$ Lyapunov orbit. These transfers possess similarly low maneuver requirements and flight times that differ by 6-7 days with each additional revolution. However, the *k*-NN graph constructed using the modified Hausdorff distance (defined in Eq. 19 to assess geometric similarity) incorrectly groups all three transfers together, requiring manual separation. Future work to address this issue includes modifying this distance measure to capture the number of revolutions performed along a transfer as well as additional geometric properties.

### Spatial Transfers from an $L_1$ to $L_2$ Northern Halo Orbit

The primitive-based trajectory design framework is used to construct spatial transfers from an $L_1$ northern halo orbit at $C_J \approx 3.0635$ to an $L_2$ northern halo orbit at $C_J \approx 3.0669$ in the Earth-Moon CR3BP with impulsive maneuvers. In this scenario, Poincaré maps capturing spatial motion at high energy levels may be difficult to analyze due to the complexity of the solution space and higher-dimensional description of the map crossings. As a result, it may be challenging to use existing dynamical systems techniques alone to construct point solutions and explore the broader design space spanned by geometrically dissimilar solutions. Thus, this challenging scenario supports demonstration of the utility of the presented motion primitive framework for trajectory design in a multi-body system.

To construct a motion primitive graph in this scenario, the high-level itinerary graph is designed to possess the same structure as in Fig. 10 but uses primitives of the selected northern halo orbit families and their stable and unstable manifolds. Consistent with the approach in Sect. 3.1, each manifold is generated for up to 15 apses relative to the Moon and sampled to produce shorter arcs that span up to 4 apses relative to the Moon. The entire set of motion primitives generated to summarize arcs along these stable and unstable manifolds are plotted in Appendix A and all components of the motion primitive library are listed in Table 3. To construct the motion primitive graph, the following configuration parameters are also specified: $k = 15$, $\alpha _{\text {pos}} = 100$, and $\alpha _{\text {vel}} = 1$. These selections place a much stronger emphasis on position discontinuities between primitives compared to the previous transfer design scenario, consistent with an observed increase in sensitivity for higher energy spatial transfers with close lunar passes. Additionally, the set of representative trajectories associated with each motion primitive is incorporated into the edge weight computations and the average edge weight is used to evaluate the quality of each primitive sequence.

**Table 3:** Motion primitives in the library for the $L_1$ to $L_2$ northern halo orbit transfer design scenario

Fundamental solution	Number of primitives	Approx. $C_J$
$L_1$ northern halo orbit	1	3.0635
$L_1$ northern halo orbit unstable manifold	198	3.0635
$L_1$ northern halo orbit stable manifold	194	3.0635
$L_2$ northern halo orbit	1	3.0669
$L_2$ northern halo orbit unstable manifold	226	3.0669
$L_2$ northern halo orbit stable manifold	223	3.0669

The motion primitive graph is searched to produce unique sequences of four, five, and six primitives that each form an initial guess for a transfer from the $L_1$ northern halo orbit to the $L_2$ northern halo orbit. There are a total of 331, 19,764, and 1,148,147 primitive sequences from the initial node to the target node in the graph consisting of four, five, and six primitives, respectively. However, using the filtering process presented in Sect. 3.3, the $Q = 10$ top-ranked sequences that begin with a unique primitive are examined for each path length. A smaller value of *Q* is selected for this scenario compared to the previous example because the quality of the initial guesses degrades more significantly as additional sequences are considered. This approach produces 30 unique primitive sequences that are each refined to produce an initial guess.

Each initial guess is corrected with several unconstrained impulsive maneuvers distributed along the transfer using the same maneuver placement scheme as in the previous example. All 30 primitive-based initial guesses are successfully corrected using $\varvec{w}_{{{\textbf {opt}}}} = [0.9, 0.1]$ to produce continuous transfers from the desired initial $L_1$ northern halo orbit to the target $L_2$ northern halo orbit. However, six of the corrected transfers impact a spherical approximation of the Moon. Continuation is then used to gradually vary the weights of the multi-objective optimization problem in Sect. 3.5 from $\varvec{w_1} = [0.9, 0.1]$ to $\varvec{w_2} = [0.1, 0.9]$. Following this process, only 27 of these transfers are successfully corrected with $\varvec{w}_{{{\textbf {opt}}}} = \varvec{w_2}$.

**Fig. 14:**
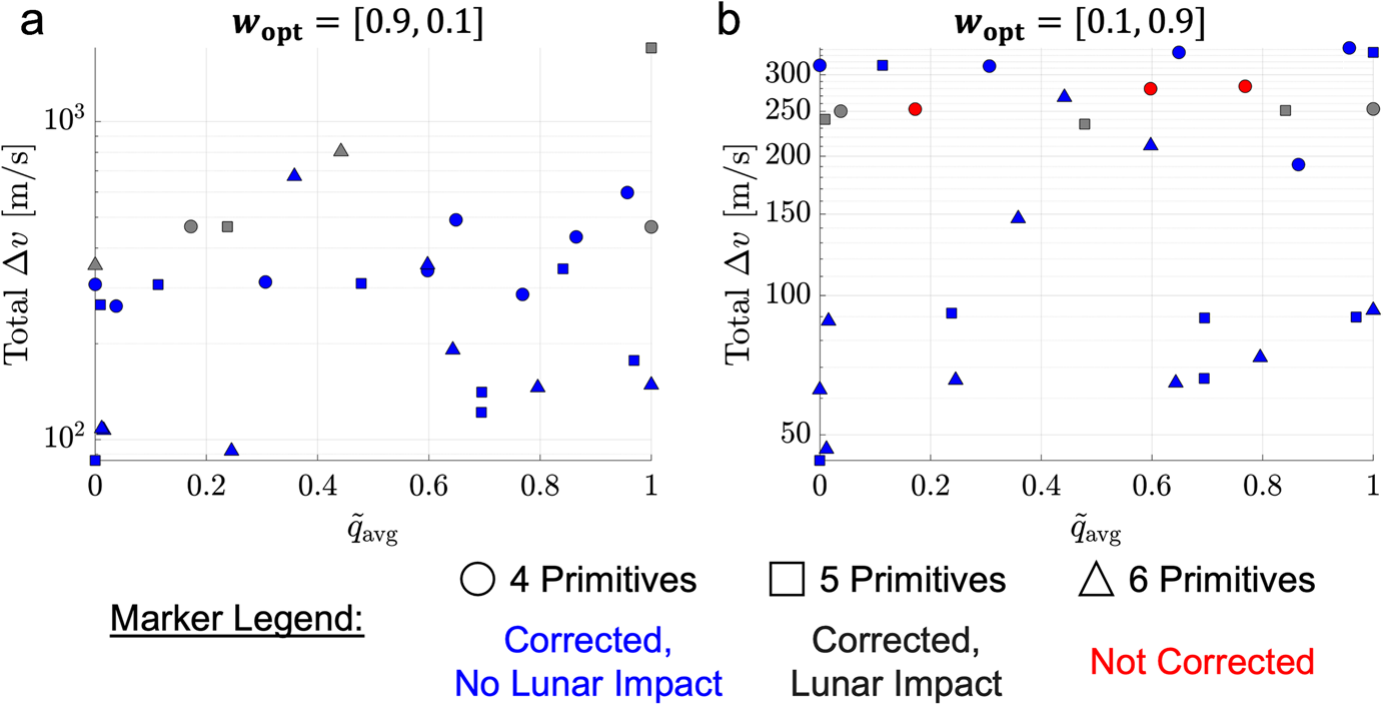
Total $\Delta v$ of transfers computed from an $L_1$ northern halo orbit to an $L_2$ northern halo orbit in the Earth-Moon CR3BP as a function of ${\tilde{q}}_{\text {avg}}$ using (a) $\varvec{w}_{{{\textbf {opt}}}} = [0.9, 0.1]$ and (b) $\varvec{w}_{{{\textbf {opt}}}} = [0.1, 0.9]$

An initial summary of the transfers that solve the multi-objective optimization problem with each value of $\varvec{w}_{{{\textbf {opt}}}}$ is presented. In Fig. 14a, the total $\Delta v$ of each of the 30 transfers that is corrected with $\varvec{w}_{{{\textbf {opt}}}} = \varvec{w_1}$ is displayed with respect to the normalized average potential for sequential composability, ${\tilde{q}}_{\text {avg}}$, of its initial guess; this figure uses the same configuration as Fig. 11a. In Fig. 14b, however, this information is presented for the 27 transfers that are corrected with $\varvec{w}_{{{\textbf {opt}}}} = \varvec{w_2}$ to prioritize minimizing maneuver requirements. Across the set of 30 transfers computed with $\varvec{w}_{{{\textbf {opt}}}} = \varvec{w_1}$ to emphasize recovering transfers that are geometrically similar to their initial guesses, the total $\Delta v$ ranges from $86.05 \hspace{4pt} \text {m/s}$ to $1705.78 \hspace{4pt} \text {m/s}$. However, when prioritizing minimizing maneuver requirements, the 27 transfers corrected with $\varvec{w}_{{{\textbf {opt}}}} = \varvec{w_2}$ require a total $\Delta v$ ranging from $44.06 \hspace{4pt} \text {m/s}$ to $342.99 \hspace{4pt} \text {m/s}$. Furthermore, the transfers summarized in Fig. 14b no longer exhibit a clear correlation between ${\tilde{q}}_{\text {avg}}$ and the total $\Delta v$ requirements compared to the planar Lyapunov orbit transfers presented in Sect. 4.1.

**Fig. 15:**
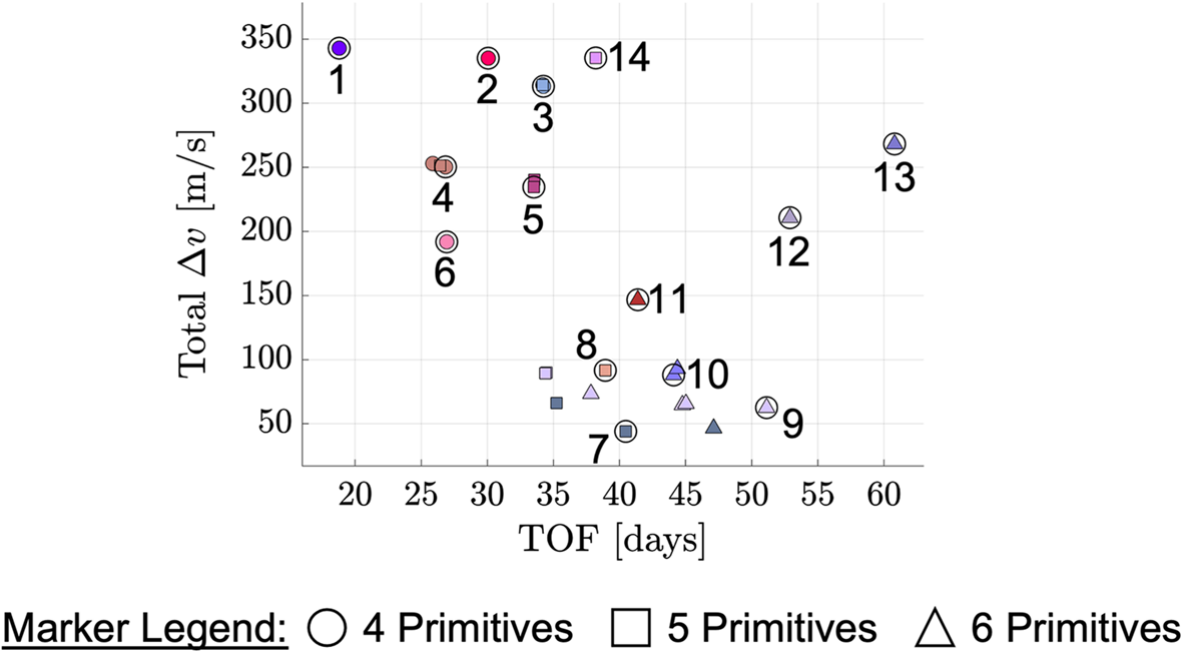
Total $\Delta v$ and TOF of transfers computed from an $L_1$ northern halo orbit to an $L_2$ northern halo orbit in the Earth-Moon CR3BP using $\varvec{w}_{{{\textbf {opt}}}} = [0.1, 0.9]$, where geometrically similar transfers are denoted in the same color and the minimum $\Delta v$ solution for each transfer geometry is highlighted

The 27 corrected transfers that prioritize minimizing maneuver requirements are grouped based on geometry to extract the distinct types of transfers that connect the selected northern halo orbits. When applying the *k*-NN approach described in Sect. 3.6, $k = 2$ is selected empirically and produces 14 groups of geometrically distinct transfers. The resulting properties of each transfer are plotted in Fig. 15 using the same configuration as Fig. 12 where the minimum $\Delta v$ solution in each group is circled and numbered. These transfers require flight times ranging from 18.79 days to 60.80 days and total $\Delta v$ requirements ranging from $44.06 \hspace{4pt} \text {m/s}$ to $342.99 \hspace{4pt} \text {m/s}$ when placing more emphasis on recovering maneuver-efficient transfers.

**Fig. 16:**
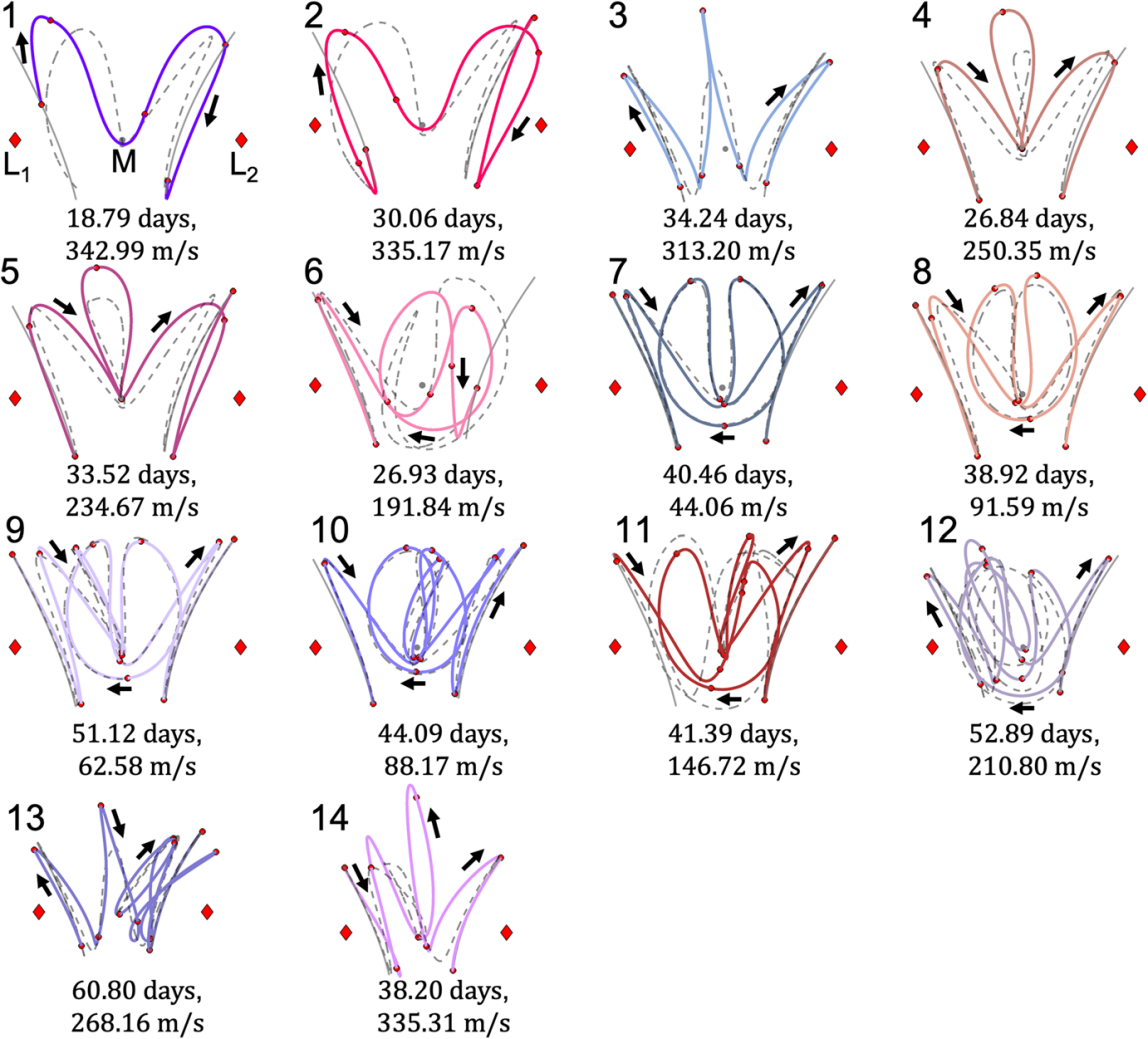
Transfers with distinct geometries computed from primitive-based initial guesses between an $L_1$ and $L_2$ northern halo orbit displayed as a projection onto the *xz*-plane of the rotating frame in the Earth-Moon CR3BP

The minimum $\Delta v$ transfer in each group is visualized in the configuration space. Specifically, Fig. 16 displays each transfer as a projection onto the *xz*-plane of the Earth-Moon rotating frame. In this figure, each corrected transfer geometrically resembles its initial guess (dashed gray) but with more noticeable deviations than in the planar transfers constructed in Sect. 4.1. Furthermore, Transfers 1-14 all exhibit distinct transfer geometries in the vicinity of the Moon with several close approaches and apolunes at high *z*-amplitudes above the plane of the primaries. The exception is Transfers 7 and 8 which could potentially be combined into a single group because these transfers exhibit only slight differences in geometry during the departure, transit, and arrival phases of the itinerary. As a comparison, these two transfers (7 and 8) geometrically resemble a 51.2 day transfer computed by Haapala between two northern halo orbits at similar energy levels, but with a lower total maneuver magnitude of 11.9 m/s [13]; this difference is likely due to alternative corrections problem formulations, an alternative number and location of maneuvers, and the explicit use of manifold arcs that gradually approach or depart each periodic orbit. Nevertheless, the recovered transfers demonstrate the capability to achieve a significant reduction in total maneuver magnitude while still preserving the approximate geometry of a coarsely-constructed primitive-based initial guess.

## Conclusion

Motion primitives, defined as the fundamental building blocks of motion, are used to develop an initial guess construction framework for spacecraft trajectories in the Earth-Moon CR3BP. First, a library of motion primitives is generated by using clustering to summarize periodic orbit families and arcs along stable/unstable manifolds. Then, a graph is constructed to capture the potential for sequential composability of motion primitives in this library and, therefore, offer a discrete representation of part of the solution space. Searching this graph produces sequences of motion primitives that support coarsely constructing initial guesses with distinct geometries. Finally, each primitive sequence is refined and corrected using direct collocation and multi-objective optimization to produce transfers that balance geometrically resembling the primitive-based initial guess with reducing maneuver requirements.

The primitive-based initial guess construction framework is demonstrated by computing a variety of transfers in the Earth-Moon CR3BP between an $L_1$ and $L_2$ Lyapunov orbit and an $L_1$ and $L_2$ northern halo orbit. In each scenario, unique primitive sequences and numerical continuation lead to the recovery of a set of transfers with a variety of distinct geometries, flight times, and maneuver requirements. These examples demonstrate that motion primitives can support initial guess construction for spacecraft trajectories in the CR3BP and rapid exploration of the associated design space.

## Figures and Tables

**Fig. 17:**
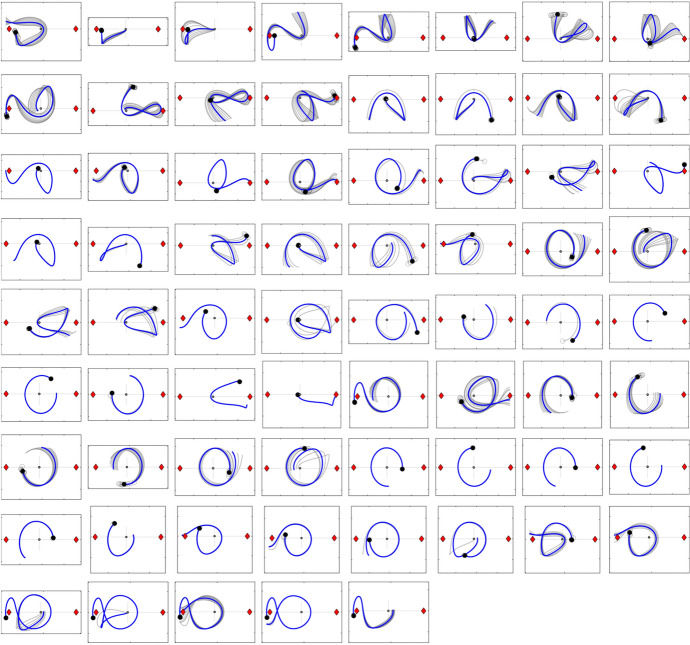
Motion primitives (blue) and the associated sets of representative trajectories (gray) computed from an $L_1$ Lyapunov orbit unstable half-manifold in the Earth-Moon CR3BP; trajectories are displayed in the *xy*-plane of the rotating frame, $L_1$ ($L_2$) is marked with a red diamond on the left (right) in each subfigure, the Moon is plotted to scale as a gray circle, the initial position of each primitive is denoted with a filled black circle, and the initial position of each representative trajectory is denoted with an empty black circle

**Fig. 18:**
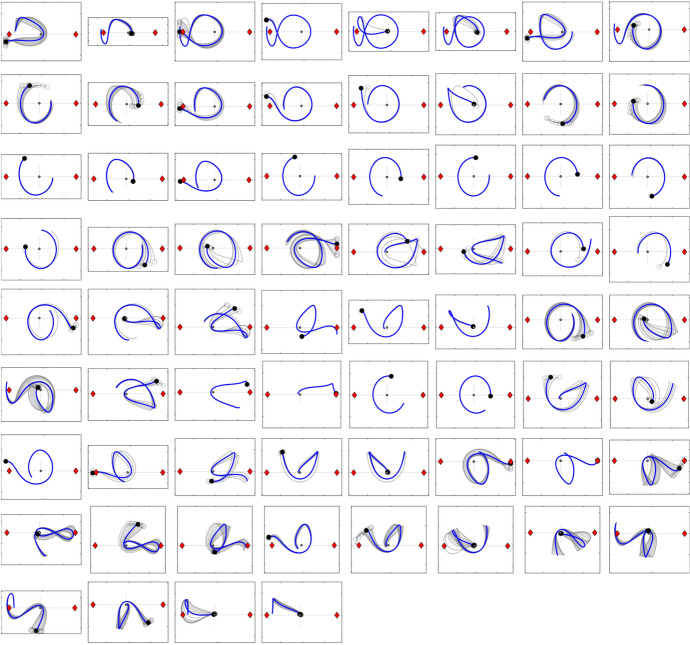
Motion primitives (blue) and the associated sets of representative trajectories (gray) computed from an $L_1$ Lyapunov orbit stable half-manifold in the Earth-Moon CR3BP; trajectories are displayed in the *xy*-plane of the rotating frame, $L_1$ ($L_2$) is marked with a red diamond on the left (right) in each subfigure, the Moon is plotted to scale as a gray circle, the initial position of each primitive is denoted with a filled black circle, and the initial position of each representative trajectory is denoted with an empty black circle

**Fig. 19:**
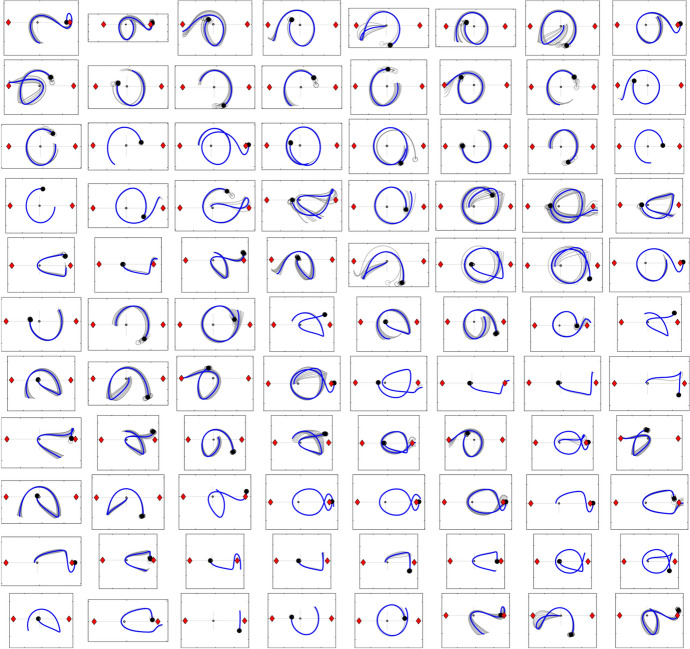
Motion primitives (blue) and the associated sets of representative trajectories (gray) computed from an $L_2$ Lyapunov orbit unstable half-manifold in the Earth-Moon CR3BP; trajectories are displayed in the *xy*-plane of the rotating frame, $L_1$ ($L_2$) is marked with a red diamond on the left (right) in each subfigure, the Moon is plotted to scale as a gray circle, the initial position of each primitive is denoted with a filled black circle, and the initial position of each representative trajectory is denoted with an empty black circle

**Fig. 20:**
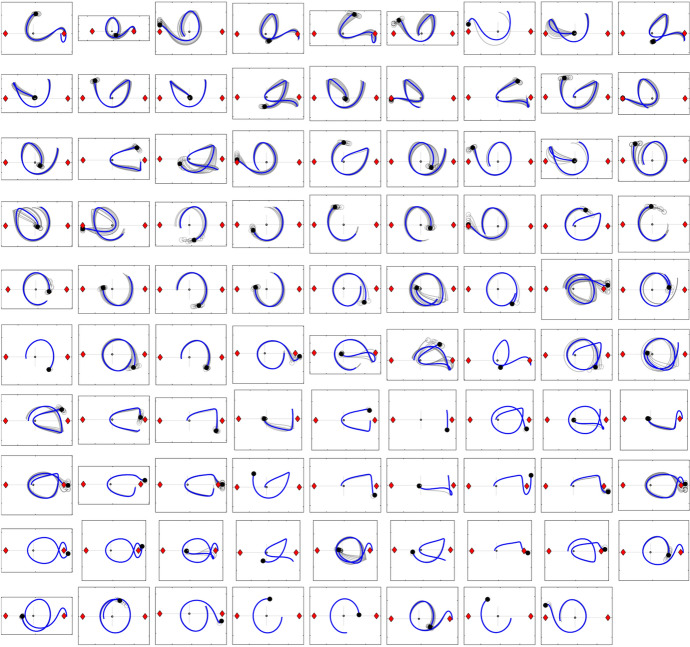
Motion primitives (blue) and the associated sets of representative trajectories (gray) computed from an $L_2$ Lyapunov orbit stable half-manifold in the Earth-Moon CR3BP; trajectories are displayed in the *xy*-plane of the rotating frame, $L_1$ ($L_2$) is marked with a red diamond on the left (right) in each subfigure, the Moon is plotted to scale as a gray circle, the initial position of each primitive is denoted with a filled black circle, and the initial position of each representative trajectory is denoted with an empty black circle

**Fig. 21:**
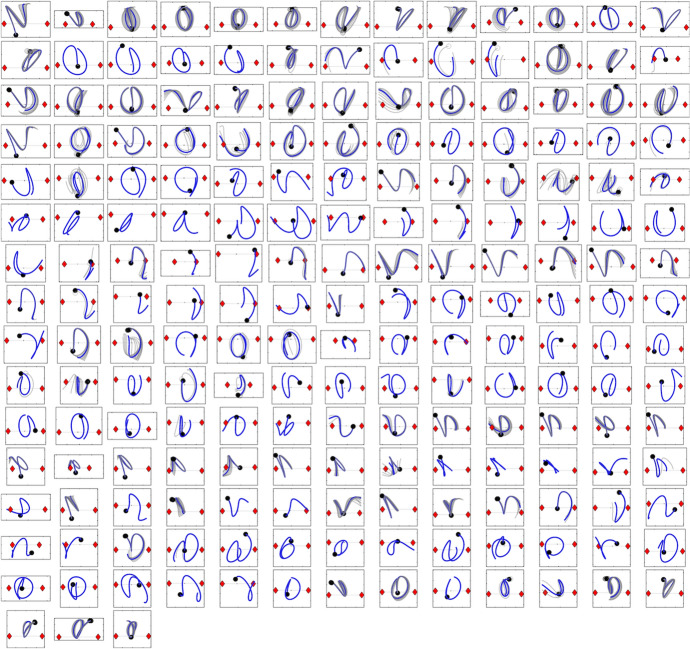
Motion primitives (blue) and the associated sets of representative trajectories (gray) computed from an $L_1$ northern halo orbit unstable half-manifold in the Earth-Moon CR3BP; trajectories are displayed as a projection onto the *xz*-plane of the rotating frame, $L_1$ ($L_2$) is marked with a red diamond on the left (right) in each subfigure, the Moon is plotted to scale as a gray sphere, the initial position of each primitive is denoted with a filled black circle, and the initial position of each representative trajectory is denoted with an empty black circle

**Fig. 22:**
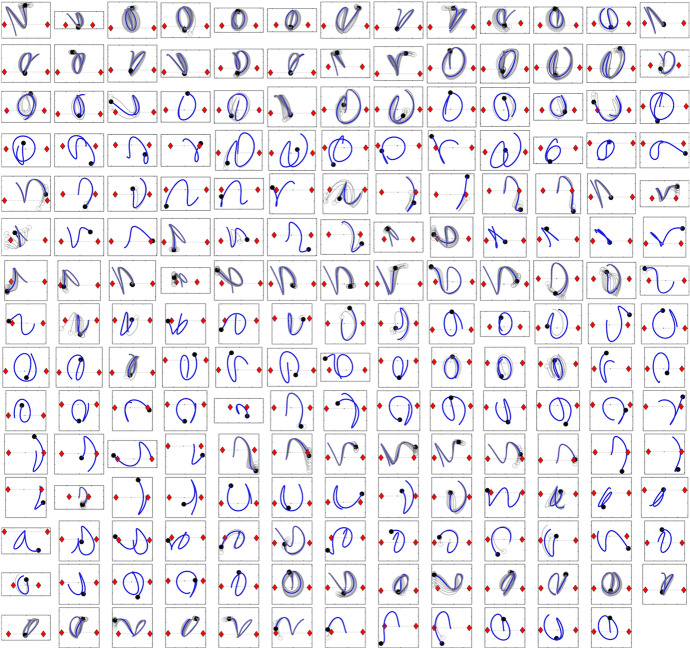
Motion primitives (blue) and the associated sets of representative trajectories (gray) computed from an $L_1$ northern halo orbit stable half-manifold in the Earth-Moon CR3BP; trajectories are displayed as a projection onto the *xz*-plane of the rotating frame, $L_1$ ($L_2$) is marked with a red diamond on the left (right) in each subfigure, the Moon is plotted to scale as a gray sphere, the initial position of each primitive is denoted with a filled black circle, and the initial position of each representative trajectory is denoted with an empty black circle

**Fig. 23:**
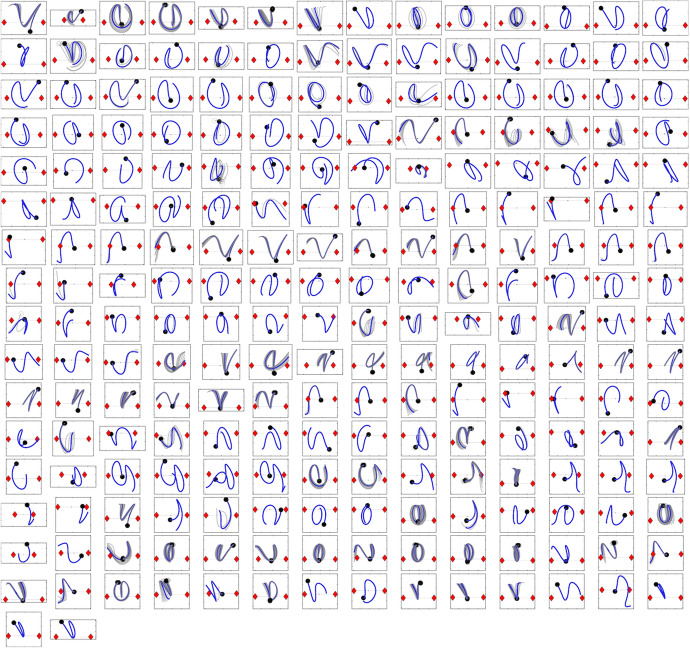
Motion primitives (blue) and the associated sets of representative trajectories (gray) computed from an $L_2$ northern halo orbit unstable half-manifold in the Earth-Moon CR3BP; trajectories are displayed as a projection onto the *xz*-plane of the rotating frame, $L_1$ ($L_2$) is marked with a red diamond on the left (right) in each subfigure, the Moon is plotted to scale as a gray sphere, the initial position of each primitive is denoted with a filled black circle, and the initial position of each representative trajectory is denoted with an empty black circle

**Fig. 24:**
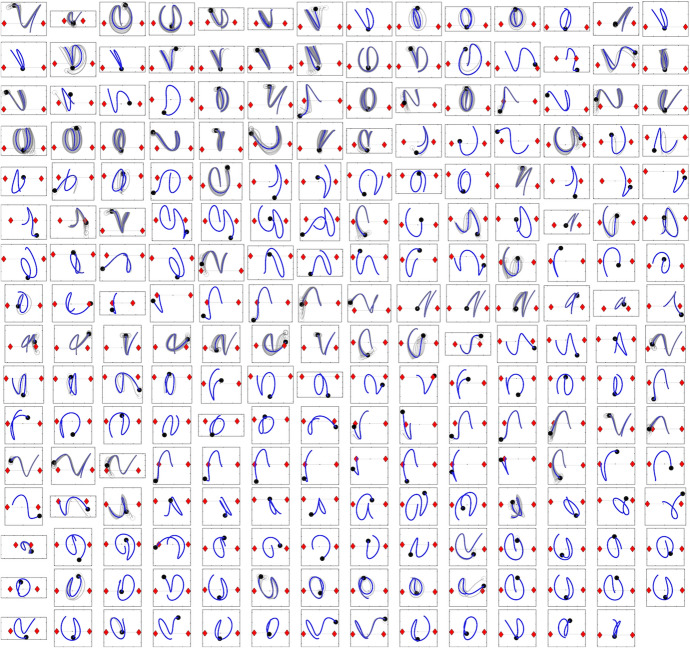
Motion primitives (blue) and the associated sets of representative trajectories (gray) computed from an $L_2$ northern halo orbit stable half-manifold in the Earth-Moon CR3BP; trajectories are displayed as a projection onto the *xz*-plane of the rotating frame, $L_1$ ($L_2$) is marked with a red diamond on the left (right) in each subfigure, the Moon is plotted to scale as a gray sphere, the initial position of each primitive is denoted with a filled black circle, and the initial position of each representative trajectory is denoted with an empty black circle

## Appendix A: Motion Primitive Library

See Fig. 17, 18, 19, 20, 21, 22, 23, 24

## Declarations

### Data Availability

No additional data or materials are provided.

### Code Availability

No additional codes are provided.
